# Reporting practices for genomic epidemiology of tuberculosis: a systematic review of the literature using STROME-ID guidelines as a benchmark

**DOI:** 10.1016/s2666-5247(20)30201-9

**Published:** 2021-03-02

**Authors:** Brianna Cheng, Marcel A Behr, Benjamin P Howden, Theodore Cohen, Robyn S Lee

**Affiliations:** Department of Epidemiology, Biostatistics and Occupational Health, McGill University, Montreal, QC, Canada; Department of Epidemiology, Biostatistics and Occupational Health, McGill University, Montreal, QC, Canada; Infectious Diseases and Immunity in Global Health Program, Research Institute of the McGill University Health Centre, McGill University, Montreal, QC, Canada; McGill International TB Centre, McGill University, Montreal, QC, Canada; The Microbiological Diagnostic Unit Public Health Laboratory, Department of Microbiology and Immunology, The University of Melbourne at The Peter Doherty Institute for Infection and Immunity, Melbourne, VIC, Australia; Yale University, New Haven, CT, USA; Epidemiology Division, Dalla Lana School of Public Health, University of Toronto, Toronto, ON, Canada; Center for Communicable Disease Dynamics, Harvard School of Public Health, Boston, MA, USA

## Abstract

**Background:**

Pathogen genomics have become increasingly important in infectious disease epidemiology and public health. The Strengthening the Reporting of Molecular Epidemiology for Infectious Diseases (STROME-ID) guidelines were developed to outline a minimum set of criteria that should be reported in genomic epidemiology studies to facilitate assessment of study quality. We evaluate such reporting practices, using tuberculosis as an example.

**Methods:**

For this systematic review, we initially searched MEDLINE, Embase Classic, and Embase on May 3, 2017, using the search terms “tuberculosis” and “genom* sequencing”. We updated this initial search on April 23, 2019, and also included a search of *bioRxiv* at this time. We included studies in English, French, or Spanish that recruited patients with microbiologically confirmed tuberculosis and used whole genome sequencing for typing of strains. Non-human studies, conference abstracts, and literature reviews were excluded. For each included study, the number and proportion of fulfilled STROME-ID criteria were recorded by two reviewers. A comparison of the mean proportion of fulfilled STROME-ID criteria before and after publication of the STROME-ID guidelines (in 2014) was done using a two-tailed *t* test. Quasi-Poisson regression and tobit regression were used to examine associations between study characteristics and the number and proportion of fulfilled STROME-ID criteria. This study was registered with PROSPERO, CRD42017064395.

**Findings:**

976 titles and abstracts were identified by our primary search, with an additional 16 studies identified in *bioRxiv*. 114 full texts (published between 2009 and 2019) were eligible for inclusion. The mean proportion of STROME-ID criteria fulfilled was 50% (SD 12; range 16–75). The proportion of criteria fulfilled was similar before and after STROME-ID publication (51% [SD 11] *vs* 46% [14], p=0·26). The number of criteria reported (among those applicable to all studies) was not associated with impact factor, h-index, country of affiliation of senior author, or sample size of isolates. Similarly, the proportion of criteria fulfilled was not associated with these characteristics, with the exception of a sample size of isolates of 277 or more (the highest quartile). In terms of reproducibility, 100 (88%) studies reported which bioinformatic tools were used, but only 33 (33%) reported corresponding version numbers. Sequencing data were available for 86 (75%) studies.

**Interpretation:**

The reporting of STROME-ID criteria in genomic epidemiology studies of tuberculosis between 2009 and 2019 was low, with implications for assessment of study quality. The considerable proportion of studies without bioinformatics version numbers or sequencing data available highlights a key concern for reproducibility.

## Introduction

Whole genome sequencing (WGS) has been increasingly used in genomic epidemiology studies. Its superior resolution compared with classical genotyping methods (eg, restriction fragment length polymorphism or mycobacterial interspersed repetitive unit-variable number tandem repeats for tuberculosis) provides the opportunity to gain new insights into transmission and evolution of drug resistance, and to potentially inform public health interventions.^1–4^ However, the ability of WGS to serve these purposes depends on the quality of the studies that use this technology. Currently, the heterogeneity of WGS bioinformatic pipelines poses challenges to the standardised reporting and interpretation of results across genomic epidemiology studies.^5,6^ Standardised reporting of data and software would further facilitate comparison of WGS-based findings, and enable researchers to assess the validity of published data.^7^

In 2007, guidelines called Strengthening the Reporting of Observational Studies in Epidemiology (STROBE) were published. These consisted of 22 criteria^8^ outlining study details that should be reported to help readers better assess quality and validity of results. In 2014, the Strengthening the Reporting of Molecular Epidemiology for Infectious Diseases (STROME-ID) guidelines were released.^9^ These extended the original 22 STROBE criteria with 20 additional criteria for reporting of genomic epidemiology studies (appendix 1 pp 14–15). In this Article, unless otherwise stated, we define STROME-ID as the combined set of STROBE and STROME-ID criteria.

The impact of the STROBE guidelines on reporting quality has been inconsistent.^10–13^ However, higher reporting quality (ie, a larger number of criteria in the guidelines being reported) has previously been associated with greater sample size^14,15^ and, to a lesser degree, with journal impact factor.^13^ To our knowledge, no previous studies have investigated factors associated with reporting quality using STROME-ID for pathogen genomic epidemiology. We systematically reviewed genomic epidemiology studies, using tuberculosis as an example, to determine the extent to which STROME-ID criteria have been reported, and whether specific study or journal characteristics were associated with reporting practices.

## Methods

### Search strategy and selection criteria

This systematic review was done according to Preferred Reporting Items for Systematic Reviews and Meta-Analyses guidelines.^16^ We initially searched MEDLINE, Embase Classic, and Embase on May 3, 2017, using the terms “tuberculosis” and “genom* sequencing”. We updated this search on April 23, 2019, and included a search of *bioRxiv*. No restrictions were placed on start date or geographic location. References of included articles were also searched manually. A detailed search strategy is described in appendix 1 (p 3).

The titles and abstracts of studies were initially screened by BC and RSL to determine whether they met inclusion criteria, which was followed by full-text review Discrepancies were resolved by discussion and third-party arbitration (TC). Eligible studies included patients with microbiologically confirmed tuberculosis and used WGS for typing of strains. Studies must have been published in English, French, or Spanish. As suggested by Field and colleagues,^9^ we considered studies to be genomic epidemiology reports if they investigated the distribution or transmission dynamics of tuberculosis across time, in a particular population, or in a geographical location in order to inform outbreaks, evaluate infection control practices, or perform surveillance. Studies were also included if they examined risk factors for transmission or if they distinguished between recurrent cases of tuberculosis as relapse or reinfection. If studies described the evolution of tuberculosis, drug resistance, or both, or if they identified and classified new tuberculosis strains or lineages, they were included. Finally, studies were included if they investigated the association between strain types or mutations and clinical outcomes (eg, death, treatment failure, or relapse).

We excluded non-human studies, studies that were exclusively experimental (eg, in-vitro or in-vivo animal studies), or those that were purely diagnostic. Conference abstracts, editorials, and literature reviews were also excluded. A full list of exclusion criteria is provided in appendix 1 (p 3).

### Data analysis

Each STROME-ID variable was assessed and scored as complete or incomplete. Some variables, evaluated by BC with consideration of the study design, were scored as not applicable. The number and proportion of fulfilled STROME-ID criteria were tabulated for each article, with the denominator for the proportions excluding criteria that were not applicable (eg, specific to a different study design). In addition, we analysed whether certain study or journal characteristics were associated with the number and proportion of fulfilled STROME-ID criteria, which were specified a priori. These were the journal impact factor, sample size of isolates, the geographic region of the senior author’s primary affiliation, and the h-index of the senior author (appendix 1 pp 3–4).

To assess differences in reporting after the publication of STROME-ID guidelines, the mean proportions of fulfilled criteria were compared before and after the publication date (April 1, 2014). A 6-month lag period was included to account for articles that were already in press when STROME-ID was published. Sensitivity analyses were also done using a 12-month lag period, and excluding articles published within 6 months and 12 months after STROME-ID publication. Differences in mean proportions of criteria were compared before and after publication using a two-tailed *t* test. The least and most reported STROME-ID criteria were also qualitatively assessed to explore differences between periods, excluding criteria that were not applicable for more than 20% of articles (appendix 1 pp 6–7). Finally, to evaluate potential differences in reporting according to study theme, we did a post-hoc analysis of the proportion of fulfilled STROME-ID criteria for the most common themes identified.

To examine the association between study and journal characteristics and reporting, two approaches were used. First, we used quasi-Poisson regression (to account for under-dispersion) with the number of criteria fulfilled as the dependent variable. This analysis was restricted to criteria that were applicable across all studies. Second, we used tobit regression (censored between 0 and 1) to assess the association with the proportion of criteria that were completed, including all studies in the analysis. Impact factor was used as a categorical variable (0 to <5, 5 to <10, 10 to <20, ≥20), with categories chosen based on our experience with the metric and previous studies that examined associations with impact factor.^17,18^ The sample size of isolates was categorised into quartiles due to low counts across a wide range of data (appendix 1 p 9). h-index was analysed as a linear variable.

Variables that had a p value of less than 0·20 in univariate analyses were included in the final model for each analysis. Pseudo-*R*^2^, the Akaike information criterion, and log-likelihood were calculated to assist with model selection and to evaluate fit. All analyses were done using R (version 1.1.456).

Finally, because STROME-ID aims to support transparent reporting practices,^9^ which is important for reproducibility, we investigated whether authors reported the bioinformatics tools used, along with corresponding version numbers for software, and whether studies had uploaded their genomic data to an open-access sequence archive.

This study was registered with PROSPERO, CRD42017064395.

### Role of the funding source

The funder of this study had no role in study design, data collection, data analysis, data interpretation, or writing of the report. All authors had full access to all the data in the study and had final responsibility for the decision to submit for publication.

## Results

**Figure 1:**
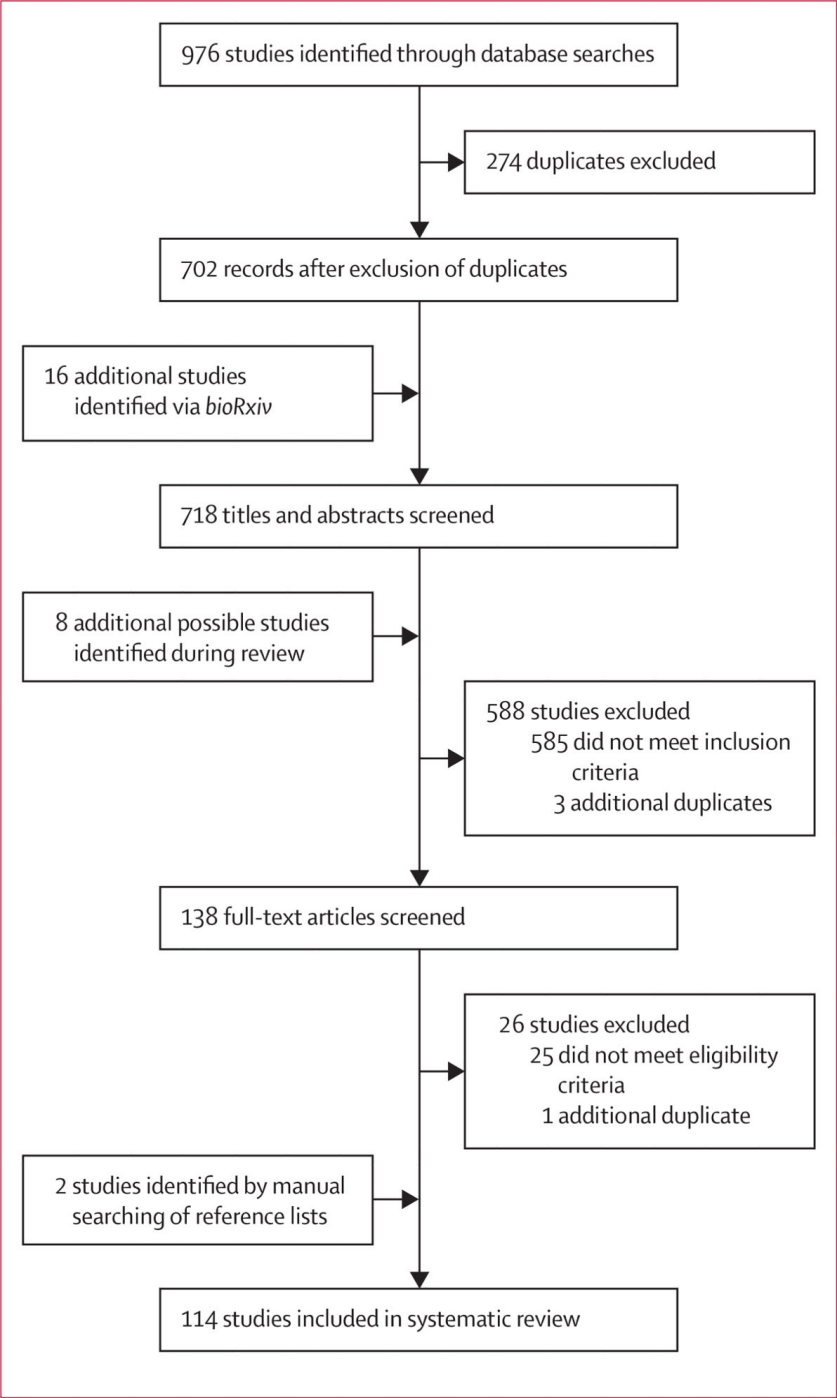
Study selection Full texts were excluded for the following reasons: conference abstract or case report (n=3), no epidemiological aims (n=12), drug resistance prediction (n=2), inadequate or no use of whole genome sequencing (n=6), did not meet inclusion criteria (n=2).

Our initial search identified 976 studies, of which 274 were duplicates and were excluded. After the addition of 16 studies identified in *bioRxiv*, 718 titles and abstracts were screened. Of these, 138 full-text articles were screened, and 114 full texts were eligible for inclusion (figure 1). 97 of 114 studies were published after STROME-ID guidelines. No studies were excluded due to language of publication. A summary of key characteristics of included studies is shown in table 1 (further detail in appendix 2).^1,19–130^ Studies were classified into four themes based on their overall aims (these themes were not mutually exclusive): transmission (n=82), evolution (n=36), strain identification (n=11), and clinical outcomes (n=2; appendix 1 p 5). The number of patients was missing for 21 (18%) articles. Impact factor was also not available for one article published during the first year of the journal (2013) and from 15 articles published in 2019 (13%).

Overall, we found that the proportion of applicable STROME-ID criteria fulfilled among the included studies ranged from 16% to 75% (mean 50% [SD 12]). There was no significant difference between the average proportion of fulfilled criteria in studies from before and after guideline publication (table 2). Both before and after guideline publication, STROME-ID 4.1 (definitions for molecular terminology; 0% before, 11% after) and STROME-ID 8.1 (methods used to detect multiple-strain infections; 6% before, 7% after) were among the least reported criteria. Across both time periods, both STROBE-3 (study objectives and hypotheses; before 94%, after 97%) and STROME-ID 3.1 (epidemiological objectives of using molecular typing; before 100%, after 95%) were among the top reported criteria. The same 15 criteria were not applicable in at least 20% of papers both before and after STROME-ID publication (appendix 1 pp 6–7); of these, 12 (80%) were from the original STROBE guidelines, and pertained to specific epidemiological study designs or statistical analyses that are less likely to be used in genomic epidemiology studies.

**Figure 2:**
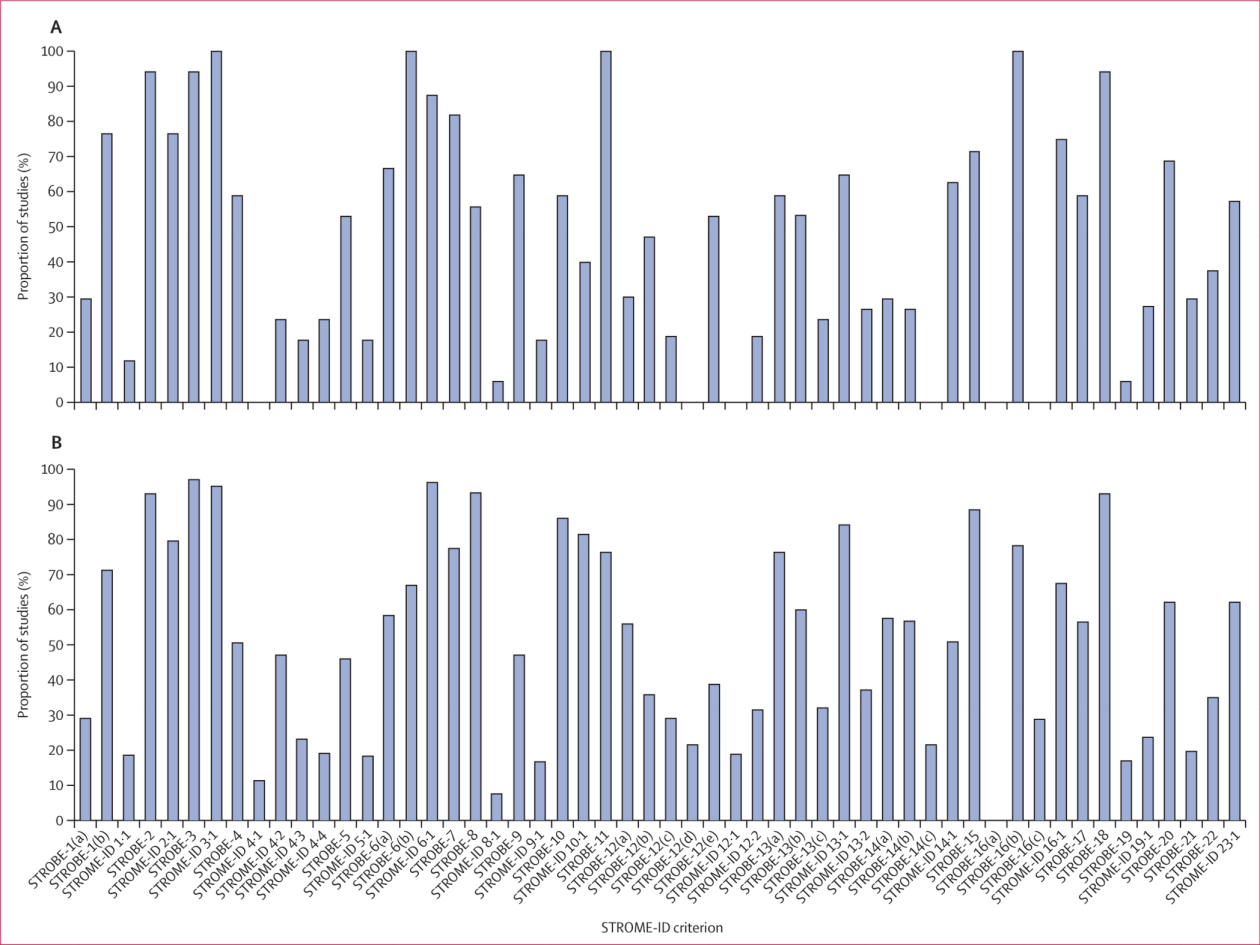
Proportion of STROME-ID criteria fulfilled before (A) and after (B) publication of the STROME-ID guidelines For this analysis, a 6-month lag period was used; studies published within 6 months of STROME-ID publication were classified as before publication instead of after publication. Definitions of the criteria are provided in appendix 1 (pp 14–15). STROBE=Strengthening the Reporting of Observational Studies in Epidemiology. STROME-ID=Strengthening the Reporting of Molecular Epidemiology for Infectious Diseases.

The average proportions of studies that fulfilled each individual STROME-ID criterion are shown in figure 2. Before STROME-ID publication (figure 2A), six STROME-ID criteria were not fulfilled by any of the included studies, whereas after publication (with a 6-month lag period; figure 2B), a single criterion, STROBE-16(a), was not completed. Similar results were found in sensitivity analyses using a 12-month lag period or excluding articles published during the 6 or 12 months after guideline publication (appendix 1 pp 10–13).

To evaluate potential differences in reporting according to study theme, we reviewed the proportion of fulfilled STROME-ID criteria among the two most common themes: transmission and evolution. Examining potential differences in reporting for transmission-only (n=67) and evolution-only (n=21) studies (ie, excluding 13 manuscripts which were classified under both of these themes), proportions of criteria reported were similar before and after publication within both themes (appendix 1 pp 17–18). The average proportions of criteria reported overall were low for both themes (51% [SD 13] for transmission-only studies, 44% [12] for evolution-only studies).

We next considered whether reporting quality was associated with specific journal and author characteristics. Because we did not detect a difference between the reporting quality before and after STROME-ID publication, we included all papers published over the entire study period for this analysis. The distribution of impact factors from all studies is shown in appendix 1 (p 8). For articles published in 2019, an evaluation of impact factors between 2013 and 2018 showed little variation across these years (appendix 1 p 16); therefore, the 2018 values were used. One paper in 2013 did not have an impact factor and was excluded from the analysis. Moreover, due to low individual country counts, we analysed author affiliation by continent. There was only one study in South America, which was subsequently combined with North America to form the category Americas (appendix 1 p 19).

Univariate and multivariate analyses for quasi-Poisson regression and tobit regression models are presented in table 3 and appendix 1 (p 20), respectively. h-index did not meet the criteria for inclusion in the full multivariate model for either quasi-Poisson or tobit regression models. There was no association between sample size of isolates, impact factor, or geographic region of the senior author, and the number of STROME-ID criteria fulfilled. Similar results were found in the multivariate tobit regression analysis, although a sample size of isolates of 277 or more was significantly associated with the proportion of criteria fulfilled (p=0·0070). 12 studies had more than one senior author; sensitivity analyses excluding these manuscripts yielded similar results (appendix 1 pp 21–22).

In terms of reporting of the bioinformatics tool used and the availability of genomic data, 100 (88%) articles reported the names of bioinformatic tools; however, only 33 (33%) of these provided version numbers for all of the tools (appendix 2). 86 (75%) papers reported accession numbers for their raw genomic data (appendix 1 p 23).

Given that genomic epidemiology studies aim to inform public health, we investigated whether any articles reported clinical or public health actions as a result of their findings. Possibly due to the retrospective nature of most of these studies, only three (3%) of included studies reported such changes; specifically, WGS results helped identify linked cases, guide tailored drug treatment based on drug-resistance analysis, and informed epidemiological investigations.^32,50,123^ Of note, one additional study reported their WGS findings to national tuberculosis surveillance programmes, but subsequent public health intervention was not possible because of the region’s political instability.^131^

## Discussion

STROME-ID was developed by an interdisciplinary team with expertise in infection control and infectious diseases,^9^ to facilitate the reporting of a minimal set of study variables that were considered critical for assessment of bias and study quality. Herein, we have used STROME-ID as the framework to evaluate the reporting and transparency of genomic epidemiology studies of tuberculosis and have explored the association between specific journal or study characteristics and reporting practices.

Publication of guidelines has previously been shown to improve reporting practices.^10,132^ Although we hypothesised that there would be differences in variables reported following the publication of STROME-ID guidelines, we found no evidence of this in the current study. On average, only around half of STROME-ID criteria were completed both before and after their publication, a finding similar to that from other systematic reviews that evaluated reporting quality after publication of STROBE.^11,12,131,133^ The proportions of criteria completed in these reviews ranged from 51·4% to 76·5%.^11,12,131,133^ Although the proportions of criteria completed before and after STROME-ID publication were similar, we note that fewer criteria were never completed in the post-publication period. However, this difference could simply be due to temporal changes, such as an increased demand for reproducibility, and could be unrelated to STROME-ID.

There could be several reasons for the observed low reporting of STROME-ID criteria. Given that only one included article specifically cited the guidelines,^123^ lack of awareness could be an issue.^134^ Previous studies have also shown that formal journal endorsement of STROBE reporting guidelines improves reporting adherence,^135,136^ but to our knowledge, no publishers require authors to follow and report adherence to STROME-ID guidelines. Other practical limitations, such as article word count and absence of online supplements, could have also influenced reporting practices. Journal support of STROME-ID is probably needed to improve reporting transparency. We also did not find any articles that completed all STROME-ID criteria, which could suggest that some of the criteria in the guidelines are too vague or difficult to complete in practice.

In terms of which criteria were less likely to be reported, we found STROME-ID criteria that concerned key definitions, methods, and potential limitations to be more poorly reported. Although it might seem trivial that the least completed STROME-ID criteria related to the defining of molecular terminology, we would argue that standardisation of basic microbiological terminology is essential to allow for clear comparisons between studies and correct interpretation of results for public health. Despite this, even in the same academic field, terms such as strain, isolate, and clone are sometimes used differently by researchers.^137^ In addition, we note that STROME-ID 8.1 (methods for detecting multi-strain infections) was also reported poorly across the entire study period. Although this criterion was investigated by some of the included papers, methods for discriminating within-host diversity using WGS data are an area of active research,^85,127^ which could explain why these were less frequently discussed.

Journal impact factor has often been used as an indicator of quality,^138^ by funding organisations,^139,140^ and even for academic promotion.^140^ However, our analyses suggest that reporting quality is not associated with impact factor, adjusting for sample size of isolates and geographic region of the senior author. Similarly, we found no association between h-index and reporting quality. These findings highlight the limitations of such indicators as correlates of the quality of scientific publications, supporting previous studies.^139,141,142^ Moreover, sample size of isolates was not found to be associated with the number of criteria completed; studies with 153–276 isolates completed a similar number of mean criteria as those with 277 or more. Although a sample size of 277 or more was associated with a higher proportion of criteria being reported, this was equivalent to less than a 10% increase compared with the reference group of less than 30 samples, and only a 2% difference from a sample size of 153–276 isolates, the adjacent category. Therefore, although this result is statistically significant, we suspect that it is not an epidemiologically meaningful difference.

In addition to STROME-ID criteria, we also investigated whether bioinformatics tools (at a minimum) were well documented in tuberculosis genomic epidemiology papers, because reproducibility is a critical concern in genomic studies.^143,144^ Although we found that articles frequently reported the name of the tool, the corresponding version number of the software was reported much less frequently, consistent with a recent analysis of RNA-seq methodology.^145^ The inclusion of version numbers is essential to evaluate bias, reproduce workflows, and compare results across studies, which, as proposed by Simoneau and colleagues,^145^ suggests the need for standardised reporting of these methodological details. Even more surprisingly, we found that nearly a quarter of studies did not provide a Sequence Read Archive or Genbank accession number for their sequencing data, with no improvement across the study period. This is problematic because it not only prevents researchers from reproducing analyses and verifying results,^146^ but in the context of infectious diseases, it can hinder public health investigations that rely on global strain depositions for genomic context or for evaluation of cross-jurisdictional transmission. We therefore suggest that data deposition should be a requirement for publication, rather than just a social norm in genomic epidemiology. However, such a change will be unlikely without collaboration (and enforcement) by funders, publishers, or both.^143^

Overall, this study has several strengths. First, it represents a comprehensive review of reporting practices in tuberculosis genomic epidemiology studies, starting with the first publication in tuberculosis genomic epidemiology in 2009,^147^ and including a search of unpublished literature. Using STROME-ID guidelines, we have identified key gaps in current reporting practices that could affect interpretation of results, adding to previous work that highlighted the implications of differences in analytic pipelines.^4^ To our knowledge, this is the first study to examine the application of STROME-ID guidelines (to tuberculosis or any other pathogen) and will serve as a template for other such investigations that employ similar genomic methods. In terms of analysis, we used a rigorous analytical approach and did numerous sensitivity analyses to assess the robustness of our results, lending further support to our inferences. Finally, in addition to STROME-ID criteria, we also examined variables related to reproducibility, highlighting that even in a field that has arguably embraced open science, a large proportion of studies continue to not share their underlying genomic data.

The study has also several limitations. First, we note that, given that the STROME-ID guidelines were only published in 2014, there may have not been enough time for widespread uptake of these reporting guidelines at the time this study was done. However, because we did not observe increased reporting practices even in 2019, 5 years after publication, we consider this to be unlikely. This view is supported by other studies suggesting low adherence to STROBE guidelines after their publication.^12,13,148^ Furthermore, because of the small number of studies in each time period, we were not able to do an analysis controlling for secular trends (eg, an interrupted time-series). However, because we did not see evidence of any such trends on visual assessment by year, this is unlikely to affect our comparison of reporting before and after guideline publication. In our regression analyses, we specifically accounted for the time-varying nature of impact factor by using the impact factor from the study’s year of publication. We also note that, as bioinformatics pipelines are not yet standardised,^4^ our review of the reporting of bioinformatics tools was qualitative and did not require adherence to a specific pipeline or set of steps. Had we required a minimum set of tools or analytic steps be reported, we expect the reproducibility would have been assessed as being even lower. Finally, we did not separate STROME-ID criteria that required multiple pieces of information (eg, STROBE-19, which required reporting of both limitations and direction of bias); thus, if the entire criterion was not met, it was assigned as incomplete. Similarly, for bioinformatics version numbers, we considered reporting to be complete only if steps were reported with versions for all included tools; there could be differences in the reporting of version numbers across different steps in the analysis.

In this comprehensive review, we systematically examined reporting quality using STROME-ID guidelines as a benchmark. We have shown that, in general, only around 50% of STROME-ID criteria were met, potentially hindering assessment of study quality. Although good reporting practices themselves do not guarantee a study is of high quality, transparency of design, methods, and results are critical for such an assessment. The scope of the current study is limited to tuberculosis, but we expect that many of these reporting and transparency issues also apply to genomic epidemiology studies of other pathogens as well. The reasons underlying the low level of reporting are unclear, although similar reporting practices have been found with other guidelines for other types of studies.^149,150^ Possible reasons include adherence to strict word limits, low author awareness or understanding of guidelines, and, possibly, resistance to change. Alternatively, these guidelines may be too difficult to implement in practice. Further study is warranted to investigate these hypotheses.

Finally, in addition to STROME-ID, we also identified key reproducibility issues in many studies, pertaining to methods of analysis and data sharing. To improve data sharing, we suggest that data deposition should be a requirement for publication of genomic epidemiology studies. This stance will require active support from journals, with real consequences for failing to meet this obligation.^145^

## Supplementary Material

1

2

## Figures and Tables

**Table 1:** Summary of included studies

	Year	Study aims	Location	Sample size of isolates	Sample size of patients	Sequencing platforms
Al-Ghafli et al^19^	2018	Elucidate transmission dynamics and describe resistance-conferring mutations	Saudi Arabia	205	NR	Illumina NextSeq
Alaridah et al^20^	2019	Compare genotype techniques to determine transmission in a low-incidence country	Sweden	100	52	Illumina HiSeq
Arandjelović et al^21^	2019	Explore countrywide transmission routes, strain dynamics, and bacterial evolution	Serbia	103	110	Illumina MiSeq, HiSeq
Arnold et al^22^	2016	Describe XDR-TB cluster in the UK	UK	4	35	NR
Auld et al^23^	2018	Determine genomic transmission links between individuals without an epidemiologic link	South Africa	342	386	Illumina MiSeq
Ayabina et al^24^	2018	Infer whether cases represent important or local transmission	Norway	129	127	Illumina MiSeq, NextSeq
Bainomugisa et al^25^	2018	Describe strains driving the epidemic and associated drug resistance mutations	Papua New Guinea (Daru Island)	100	NR	Illumina MiSeq
Bouzouita et al^26^	2019	Investigate transmission of drug-resistant strains	Tunisia	46	46	Illumina MiniSeq
Bjorn-Mortensen et al^27^	2016	Examine transmission in remote, high-incidence region	Greenland	182	182	Illumina MiSeq, HiSeq, NextSeq
Black et al^28^	2017	Distinguish between outbreak cases of relapse from reactivation in UK	UK (England)	17	25	Illumina MiSeq
Brown et al^29^	2016	Describe genomic epidemiology of subpopulations in two cities	USA	71	NR	Illumina HiSeq
Bryant et al^30^	2013	Estimate usefulness of the molecular clock to refute and affirm epidemiological links	Amsterdam, Estonia	199	199	Illumina Genome Analyzer IIx
Bui et al^31^	2019	Assess association between exposure to community settings and MDR-TB infection	Peru	59	59	NR
Cabibbe et al^32^	2018	Describe WGS-based model for tuberculosis diagnosis and surveillance	Italy	298	56	Illumina MiniSeq
Casali et al^33^	2012	Examine microevolution of Beijing strains and spread of drug resistance	Russia	2348	2348	Illumina Genome Analyzer GAII
Casali et al^34^	2014	Explore molecular mechanisms determining transmissibility and prevalence of drug-resistant strains	Russia	1000	2348	Illumina Genome Analyzer GAII, HiSeq
Casali et al^35^	2016	Compare WGS and MIRU-VNTR to resolve the transmission network within outbreak	UK (England)	344	501	Illumina HiSeq
Chatterjee et al^36^	2017	Characterise genotypic drug resistance	India	74	NR	Illumina MiSeq
Clark et al^37^	2013	Understand emergence and acquisition of MDR-TB among treated patients with tuberculosis	Uganda	51	41	Illumina HiSeq
Cohen et al^38^	2015	Describe evolution of XDR-TB	African continent	337	337	Illumina HiSeq
Comas et al^39^	2015	Describe population genomics in Africa and evolutionary origin of tuberculosis	Ethiopia	285	2151	Illumina HiSeq
Comas et al^40^	2013	Describe evolutionary history of humans and tuberculosis	46 countries	259	259	Illumina, model unspecified
Coscolla et al^41^	2015	Describe the genomic epidemiology of MDR-TB among refugees in the USA	USA	57	45	Illumina HiSeq
Dheda et al^42^	2017	Analyse transmission dynamics of patients with XDR-TB	African continent	149	237	Illumina HiSeq
Dixit et al^43^	2019	Study evolution of isolates within an MDR-TB cluster	Peru (Lima)	61	60	Illumina HiSeq
Doroshenko et al^44^	2018	Describe the epidemiological and genomic determinants of two outbreaks	Canada	75	75	Illumina HiSeq
Eldholm et al^45^	2015	Determine timeline of drug-resistance evolution during an outbreak	Argentina	252	NR	Illumina HiSeq, Miseq
Fiebig et al^46^	2017	Investigate cross-border MDR-TB transmission	Austria, Romania, Germany	10	13	Illumina MiSeq
Gardy et al^47^	2011	Describe outbreak transmission with WGS and social network analysis	Canada	36	41	Illumina Genome Analyzer II
Gautum et al^48^	2018	Describe the genomic epidemiology of tuberculosis in Tasmania	Australia (Tasmania)	18	18	Illumina MiSeq
Gautum et al^49^	2017	Analyse the genomic content of the Rangipo strain	New Zealand	9	NR	Illumina MiSeq
Genestet et al^50^	2019	Describe tracing of linked cases in an outbreak using WGS	France	14	14	Illumina MiSeq
Glynn et al^51^	2015	Assess cases attributed to transmission from close contacts	Malawi	406	1907	Illumina HiSeq
Guerra-Assunção et al^52^	2015	Conduct district-wide analysis to examine transmission over time	Malawi	1687	2332	Illumina HiSeq
Guerra-Assunção et al^53^	2015	Assess effect of different factors on the rate of recurrence due to reinfection or relapse	Malawi	1933	903	Illumina HiSeq
Gurjav et al^54^	2016	Understand local transmission in a low-incidence setting	Australia	30	1692	Ion Torrent
Guthrie et al^55^	2018	Understand transmission dynamics of paediatric tuberculosis in a low-incidence setting	Canada	49	49	Illumina HiSeq
Ho et al^56^	2018	Describe extent of transmission based on a mass-screening exercise	Singapore	10	6	Illumina, model unspecified
Holden et al^57^	2018	Describe results of an outbreak investigation	UK (England)	2	2	Illumina HiSeq
Holt et al^58^	2018	Examine transmission dynamics	Vietnam	1635	2091	Illumina HiSeq
Huang et al^59^	2019	Describe the epidemiological and drug-resistance characteristics of MDR-TB	China	357	357	Illumina HiSeq
Ioerger et al^60^	2009	Investigate the causes and evolution of drug resistance	South Africa	11	NR	Illumina GAII
Ioerger et al^61^	2010	Understand the mechanism of drug resistance among a subgroup of the Beijing strain	South Africa	14	NR	Illumina, model unspecified
Ismail et al^62^	2018	Determine drug resistance and assess criteria against putative resistance associated with variants	South Africa	391	401	Illumina MiSeq
Jajou et al^63^	2018	Analyse transmission dynamics among asylum seekers and assess precision of VNTR typing versus WGS	Netherlands	40	40	Illumina NextSeq
Jajou et al^64^	2018	Investigate if WGS more accurately predicts epidemiological links between patients than VNTR	Netherlands	535	527	Illumina HiSeq
Jiang et al^65^	2018	Determine incidence of tuberculosis in close contacts and transmission	China	4584	1765	NR
Kato-Maeda et al^66^	2018	Describe the microevolution during an outbreak of drugsusceptible tuberculosis	USA	9	11	Illumina, model unspecified
Koster et al^67^	2013	Identify genomic differences between Beijing and Manila families	USA	82	NR	Illumina MiSeq
Koster et al^68^	2019	Investigate tuberculosis transmission clusters using WGS versus VNTR typing	USA	16	15	Illumina MiSeq
Kato-Miyazawa et al^69^	2018	Characterise genomic diversity of foreign-born and Japan-born residents in Tokyo	Japan	259	91	Illumina MiSeq
Korhonen et al^70^	2015	Determine whether recurrent cases were caused by relapse versus reinfection	Finland	21	21	Illumina MiSeq
Lalor et al^71^	2016	Delineate transmission networks and investigate benefits of WGS during cluster investigation	UK (England)	22	22	Illumina MiSeq, Genome Analyzer II, HiSeq
Lanzas et al^72^	2018	Determine extent of primary acquired MDR-TB cases	South Africa	97	NR	Illumina Genome Analyzer IIx
Lee et al^73^	2015	Explore epidemiological links during an outbreak	Canada	42	933	Illumina MiSeq
Lee et al^74^	2015	Describe genomic features of an epidemiologically successful strain over time	Canada	163	NR	Illumina MiSeq
Luo et al^75^	2015	Characterise global diversity of 358 Beijing strains	China	908	NR	Illumina HiSeq
Luo et al^76^	2015	Compare VNTR and WGS to study the transmission in a highburden setting	China	32	42	Illumina HiSeq
Ma et al^77^	2015	Explore transmission dynamics of an outbreak in a boarding school	China	33	46	Ion Torrent
Macedo et al^78^	2015	Compare WGS and classical genotyping methods to determine transmission chains	Portugal	83	83	Illumina MiSeq
Madrazo-Moya et al^79^	2019	Identify drug-resistant mutations in an endemic region	Mexico	91	91	Illumina NextSeq
Mai et al^80^	2019	Examine transmission dynamics and drug resistance-conferring mutations among patient with tuberculosis and HIV coinfection	Vietnam	200	200	Illumina NextSeq
Makhado et al^81^	2018	Determine if MDR-TB strains genotypically similar to those in Eswatini were also present in South Africa	South Africa	277	277	Illumina HiSeq, MiSeq
Malm et al^82^	2018	Determine the population structure and transmission dynamics	Congo	75	211	Illumina MiSeq
Manson et al^83^	2017	Describe prevalence of strains and evolution of drug-resistance mutations	India	223	196	Illumina HiSeq
Manson et al^84^	2017	Determine acquisition timeline of MDR drug-resistance mutations	48 countries	5310	NR	Illumina, model unspecified
Martin et al^85^	2017	Use WGS data to identify within-host heterogeneity among patients in British Columbia	Canada	25	NR	Illumina HiSeq
Mehaffy et al^86^	2018	Identify transmission events associated with cases due to ON-A strain	Canada	61	57	Illumina, model unspecified
Merker et al^87^	2015	Reconstruct evolutionary history of Beijing lineage	99 countries	4987	NR	Illumina MiSeq
Merker et al^88^	2015	Analyse evolutionary history of drug resistance and transmission networks of MDR-TB isolates	Uzbekistan	277	277	Illumina MiSeq, HiSeq
Merker et al^89^	2018	Examine mutation rates in Beijing strains from regions with MDR-TB	Germany, Georgia, Uzbekistan	NR	3	Illumina, model unspecified
Mizukoshi et al^90^	2013	Describe molecular epidemiology of patients with tuberculosis living in localised area	Japan	169	169	Illumina MiSeq
Mokrousov et al^91^	2017	Describe evolutionary origin of NEW-1 family in the EuroAmerican lineage	China, Tibet, Iran, Russia, Kazakhstan	5715	NR	Illumina MiSeq
Mortimer et al^92^	2017	Characterised population genetics of known drug resistance loci	Russia, South Africa	1161	NR	Illumina HiSeq
Nelson et al^93^	2018	Evaluate XDR-TB transmission within and between municipal districts in KwaZulu-Natal	South Africa	344	344	Illumina MiSeq
Norheim et al^94^	2018	Report use of WGS to delineate an outbreak	Norway	22	24	Illumina MiSeq, NextSeq
Ocheretina et al^95^	2017	Investigate suspected outbreak of eight cases	Haiti	8	8	Illumia HiSeq
O’Neill et al^96^	2019	Reconstruct lineage-specific patterns of spread in Africa and Eurasia	51 countries	552	NR	NR
Otchere et al^97^	2018	Compare evolution of tuberculosis and influence of human migration from two lineages	Ghana	214	NR	Illumina HiSeq, NextSeq
Outhred et al^98^	2018	Clarify transmission pathways and explore the evolution of an outbreak	Australia	23	23	Illumina HiSeq
Packer et al^99^	2016	Investigate transmission within an educational institution	UK (England)	5	10	Illumina MiSeq
Panossian et al^100^	2019	Evaluate genetic makeup of tuberculosis lineages circulating in the Middle East	Lebanon	13	13	Illumina MiSeq
Parvaresh et al^101^	2018	Analyse reinfection and reactivation rates	Australia	15	18	Illumina NextSeq
Perdigão et al^102^	2018	Determine genomic diversity and microevolution of MDR-TB and XDR-TB	Portugal	56	NR	Illumina HiSeq
Pérez-Lago et al^103^	2014	Examine microevolution of tuberculosis within intrapatient and interpatient scenarios	Spain	36	NR	Ilumina HiSeq
Regmi et al^104^	2014	Investigate outbreak of MDR-TB	Thailand	64	148	Illumina HiSeq
Roetzer et al^1^	2015	Identify outbreak-related transmission chains	Germany	86	86	Illumina, model unspecified
Roycroft et al^105^	2013	Examine acquisition and spread of MDR-TB	Ireland	42	41	Illumina MiSeq
Ruesen et al^106^	2018	Examine association between tuberculosis genotype and susceptibility to tuberculosis meningitis	Indonesia	106	322	Illumina HiSeq
Rutaihwa et al^107^	2018	Determine geographical origin of Beijing strain and spread across Africa	Africa	781	781	Illumina HiSeq
Saelans et al^108^	2019	Assess distribution of Beijing lineage	Guatemala	5	5	Illumina HiSeq, MiSeq
Satta et al^109^	2015	Examine genetic variation of outbreak samples	UK (England)	16	NR	Illumina HiSeq
Schürch et al^110^	2016	Use WGS to study epidemiology of an outbreak	Netherlands	3	NR	Genome Sequencer
Senghore et al^111^	2010	Understand epidemiology and genetics of MDR-TB	Nigeria	63	5	Illumina MiSeq
Séraphin et al^112^	2017	Define recent transmission clusters and timing of transmission	USA	21	82	Illumina MiSeq
Shah et al^113^	2018	Describe population-level transmission of XDR-TB	South Africa	298	404	Illumina MiSeq
Smit et al^114^	2017	Describe outbreak using WGS and IGRA	Finland	12	14	NR
Sobkowiak et al^115^	2018	Assess prevalence of mixed infection and correlation with patient characteristics and outcomes	Malawi, Portugal	48	10	Illumina HiSeq, MiSeq
Stucki et al^116^	2018	Study outbreak dynamics	Switzerland	69	68	Illumina, model unspecified
Stucki et al^117^	2015	Assess transmission among Swiss-born and foreign-born patients with tuberculosis	Switzerland	90	93	Illumina HiSeq, MiSeq, NextSeq
Stucki et al^118^	2016	Understand global population structure of lineage 4 and its evolution	100 countries	293	NR	Illumina MiSeq, HiSeq2000/250, NextSeq
Tyler et al^119^	2016	Characterise genomic diversity of outbreak clusters	Canada	233	NR	Illumina NextSeq
Vaziri et al^120^	2017	Explore drug resistance and transmission dynamics	Iran	38	892	Illumina NextSeq
Walker et al^121^	2019	Estimate genetic diversity of related strains and investigate community outbreaks	England	390	254	Illumina HiSeq
Walker et al^122^	2013	Explore epidemiology of transmission	England	247	269	Illumina HiSeq
Walker et al^123^	2014	Describe origin of transmission cluster	Germany, Switzerland, France, England, Somalia, Ethiopia, Eritrea	58	29	Illumina, model unspecified, Ion Torrent
Winglee et al^124^	2018	Understand geographic distribution of lineages 5 and 6	Mali	92	NR	Illumina, model unspecified
Witney et al^125^	2016	Determine proportion of cases attributable to relapse and reinfection	South Africa, Zimbabwe, Botswana, Zambia	36	51	Illumina HiSeq
Wollenberg et al^126^	2017	Understand evolution of MDR-TB and XDR-TB	Belarus	138	97	Illumina HiSeq
Wyllie et al^127^	2017	Determine proportion of linked tuberculosis isolates that are closely genomically related	England	1999	1999	Illumina MiSeq
Yang et al^128^	2018	Assess transmission of MDR-TB and identify transmission risk factors	China	324	324	llumina Hiseq
Yang et al^129^	2017	Describe transmission dynamics in an urban setting	China	218	NR	Illumina HiSeq
Yimer et al^130^	2018	Identify genomic features of lineage 7 strains	Ethiopia	30	NR	Illumina MiSeq

NR=not reported. XDR-TB=extensively drug-resistant tuberculosis. MDR-TB=multidrug-resistant tuberculosis. WGS=whole genome sequencing. MIRU-VNTR=mycobacterial interspersed repetitive unit-variable number tandem repeats. VNTR=variable number tandem repeats. IGRA=interferon γ release assay.

**Table 2:** Mean proportions of STROME-ID criteria fulfilled before and after guideline publication

	Proportion of criteria fulfilled before STROME-ID publication (%)	Proportion of criteria fulfilled after STROME-ID publication (%)	p value
6-month lag period*	51% (11)	46% (14)	0·26
12-month lag period*	48% (14)	51% (11)	0·52
6-month exclusion period†	46% (14)	46% (14)	0·98
12-month exclusion period†	48% (14)	49% (14)	0·71

Data are mean (SD). STROME-ID=Strengthening the Reporting of Molecular Epidemiology for Infectious Diseases.

* For these analyses, studies published within either 6 or 12 months of STROME-ID publication were classified as before publication instead of after publication (ie, we assumed that authors might not have seen the guidelines or had the opportunity to incorporate them within the first 6 or 12 months).

† For these analyses, papers published within 6 or 12 months of STROME-ID publication were excluded from the analysis altogether.

**Table 3:** Quasi-Poisson univariate and multivariate analyses of study characteristics

	Univariate analysis		Multivariate analysis	
	IRR (95% CI)	p value	IRR (95% CI)	p value
Impact factor of journal				
0 to <5	1 (ref)	··	1 (ref)	··
5 to <10	1·10 (1·00–1·21)	0·062	1·09 (0·98–1·22)	0·11
10 to <20	1·20 (1·03–1·38)	0·020	1·18 (1·00–1·39)	0·055
≥20	1·13 (1·00–1·28)	0·049	1·11 (0·97–1·28)	0·14
h-index	1·00 (1·00–1·00)	0·37	NA	NA
Continent of senior author				
Americas*	1 (ref)	··	1 (ref)	··
Africa	0·97 (0·79–1·18)	0·79	0·98 (0·80–1·19)	0·83
Asia	0·93 (0·81–1·08)	0·37	0·96 (0·30–1·12)	0·62
Europe	0·93 (0·84–1·02)	0·13	0·92 (0·83–1·01)	0·090
Oceania	0·91 (0·76–1·09)	0·30	0·95 (0·79–1·14)	0·60
Sample size of isolates				
<30	1 (ref)	··	1 (ref)	··
30–152	1·03 (0·92–1·15)	0·65	1·00 (0·89–1·13)	0·97
153–276	1·05 (0·90–1·21)	0·53	1·01 (0·86–1·18)	0·95
≥277	1·11 (0·99–1·25)	0·088	1·04 (0·91–1·19)	0·55

IRR=incidence rate ratio. NA=not applicable.

* North America and South America were combined because only one study was from South America.

## Research in context

### Evidence before this study

Pathogen genomics are playing an increasingly important role in infectious disease epidemiology and public health. However, the ability of genome sequencing to inform interventions depends on the quality of the studies that use this technology. In 2014, guidelines called the Strengthening the Reporting of Molecular Epidemiology for Infectious Diseases (STROME-ID) were published by a team of experts in the field, to provide authors with a minimum set of criteria for reporting and to help readers assess the validity of study methodology and results. To date, however, overall reporting practices of genomic epidemiology studies have not been evaluated with STROME-ID. Evidence of the impact of reporting guidelines on reporting practices in other fields is mixed.

### Added value of this study

In this study, we evaluated reporting practices of genomic epidemiology studies of tuberculosis using the STROME-ID guidelines as our benchmark. Overall, we found that reporting quality was low; the mean proportion of STROME-ID criteria fulfilled was only 50% (SD 12). There was no significant difference in reporting before versus after STROME-ID publication, nor did reporting appear to be associated with impact factor, h-index, geographical region of the senior author, or with the number of isolates included in the study. We also examined several important considerations for reproducibility of these studies. We found that, although 88% of studies reported which bioinformatic tools were used, only a third reported corresponding version numbers, and less than 80% of studies had made pathogen sequencing data available.

### Implications of all the available evidence

Detailed reporting of study methodology is critical to properly evaluate quality and determine how (and whether) results can inform public health interventions. Similarly, open sharing of pathogen genomic data is crucial for reproducibility of results, and as a resource for the greater scientific community. Our study suggests reporting practices in genomic epidemiology studies of tuberculosis require considerable improvement to meet guidelines. We would anticipate that many of the reporting and transparency issues identified here also apply to genomic epidemiology studies of other pathogens. We suggest that active support from scientific journals might be essential in addressing these crucial issues.
