# Supplementary material for: Reporting practices for genomic epidemiology of tuberculosis: a systematic review of the literature using STROME-ID guidelines as a benchmark
Source: Lancet Microbe. Author manuscript; Available in PMC 2021 Apr 9. (PMC8034592; doi:10.1016/s2666-5247(20)30201-9)
Supplement: 1 [file NIHMS1681620-supplement-1.pdf]

THE LANCET
Microbe

Supplementary appendix

This appendix formed part of the original submission and has been peer reviewed. We post it as supplied by the authors.

Supplement to: Cheng B, Behr MA, Howden BP, CohenT, Lee RS. Reporting practices for genomic epidemiology of tuberculosis: a systematic review of the literature using STROME-ID guidelines as a benchmark. *Lancet Microbe* 2021; **2:** e115–29.

Whole genome sequencing for epidemiological studies of tuberculosis: a systematic review of reporting practices using the 'Strengthening the Reporting of Molecular Epidemiology for Infectious Diseases' (STROME-ID) guidelines as a benchmark

Cheng B[1], Behr MA[1, 2], Howden BP[3], Cohen T[4], Lee RS[5,6]*

[1]McGill University, Department of Epidemiology, Biostatistics and Occupational Health, Montreal, Canada
[2]Infectious Diseases and Immunity in Global Health Program, Research Institute of the McGill University Health Centre, Montreal, Quebec; McGill International TB Centre, Montreal, Quebec, Canada
3The Microbiological Diagnostic Unit Public Health Laboratory, Department of Microbiology and Immunology, The University of Melbourne at The Peter Doherty Institute for Infection and Immunity, Melbourne, Australia
[4]Yale University, New Haven, United States of America
[5]University of Toronto, Dalla Lana School of Public Health, Epidemiology Division, Toronto, Canada
[6]Harvard School of Public Health, Center for Communicable Disease Dynamics, Boston, United States of America

SUPPLEMENTAL MATERIAL

Table of Contents

Supplemental Methods *3*

Supplemental Results *5*

Supplemental Figures *6*

Supplemental Figure 1. Count of "not applicable" papers per STROME-ID criterion, pre-publication........6

Supplemental Figure 2. Count of "not applicable" papers per STROME-ID criterion, post-publication........7

Supplemental Figure 3. Distribution of impact factors for included papers........8

Supplemental Figure 4. Distribution of sample size of isolates in included papers........9

Supplemental Figure 5. Proportion of STROME-ID criteria met post-publication, excluding articles from the first 6-months following guideline publication........10

Supplemental Figure 6. Proportion of STROME-ID criteria met with 12-month lag pre-publication. 11

Supplemental Figure 7. Proportion of STROME-ID criteria met with 12-month lag post-publication........12

Supplemental Tables *14*

Supplemental Table 1. STROME-ID criteria, adapted from Field et al.[2] 14

Supplemental Table 2. Standard deviation of journal IF from 2013-2018, shown for the journals corresponding to an article published in 2019. 16

Supplemental Table 5. Count of papers per continent of senior author's primary affiliation........19

Supplemental Table 6. Univariate and multivariate tobit analysis of IF, HI, Continent, and SS........20

Supplemental Table 7. Sensitivity univariate and multivariate analysis for quasi-Poisson, excluding twelve papers with >1 senior author........21

Supplemental Table 8. Sensitivity univariate and multivariate analysis for tobit regression, excluding twelve papers with >1 senior author........22

Supplemental Table 9. Number of papers with unavailable raw genomic data. 23

References *24*

Supplemental Methods

Search strategy

This study is registered on PROSPERO (CRD42017064395) and followed Preferred Reporting Items for Systematic Reviews and Meta-Analyses (PRISMA) guidelines.[1] We initially searched MEDLINE, Embase Classic and Embase on May 3, 2017 using the terms "tuberculosis" and "genom* sequencing". We then updated this search on April 23, 2019. No restrictions were placed on the start date or geographic location. We also systematically searched the pre-print server bioRxiv. References of included articles were also hand-searched to ensure no eligible articles were missed.

Inclusion and exclusion criteria

To be eligible for inclusion, studies needed to include patients with microbiologically-confirmed TB and needed to have used WGS for typing of strains. Studies must have been published in English, French or Spanish. As suggested by Field et al.,[2] we considered studies to be genomic epidemiology papers if they investigated the distribution or transmission dynamics of TB across time, a particular population, or a geographic location in order to inform outbreaks, evaluate infection control practices or perform surveillance. Studies were also included if they examined risk factors for transmission (e.g., clustering), or if they distinguished between recurrent cases of TB as relapse or reinfection. If studies described the evolution of TB strains and drug resistance, or if they identified and classified new TB strains or lineages, they were included as well. Finally, studies were included if they investigated the association between strain types or mutations and clinical outcomes (e.g., death, treatment failure, relapse).

We excluded non-human studies, studies that were exclusively experimental (e.g., in-vitro or in-vivo animal studies, or those that were purely diagnostic. The latter included studies where WGS was exclusively used for predicting phenotypic drug resistance, without epidemiological aims. We also excluded studies whose primary aim was to use WGS to develop a SNP-based typing method (unless the overall analysis and description of the epidemiology still relied on WGS), studies that exclusively compared typing methods, and studies with less than two patients. Conference abstracts, editorials, and literature reviews were also excluded.

Data extraction

To determine if manuscript met eligibility criteria for STROME-ID, two reviewers independently reviewed titles and abstracts (BC and RSL). Discrepancies were resolved by discussion and third-party arbitration (TC). The data extraction form was then piloted, with independent data extraction (by BC and RSL) of a random sample consisting of 5% of all eligible papers. Data extracted for these studies were compared and discussed to clarify understanding of the criteria and any discrepancies, prior to extraction for the remaining articles. One reviewer (BC) was responsible for data extraction based on STROME-ID criteria, as well as additional variables of interest (specified *a priori*), including whether the bioinformatic tools used were reported (along with corresponding version numbers) and whether WGS sequencing data were made openly available, to assess reproducibility. All accession numbers were checked to confirm that the raw data was uploaded for papers that reported sequence accession numbers. A second reviewer (RSL) independently checked a random sample consisting of 5% of all eligible papers; data extraction for these papers was compared between BC and RSL prior to data extraction for the remaining articles, with discussion to clarify any discrepancies.

Following data extraction, overall themes of the articles were synthesized and described. Each STROME-ID variable was assessed, and scored as 'complete' or 'incomplete' (or assigned 'not applicable', where appropriate). The number of STROME-ID criteria and proportion of those out of all criteria were then tabulated for each article, with the denominator for the proportions excluding criteria that were not applicable (e.g., specific to a different study design).

In addition to this, we analyzed whether certain study characteristics were associated with the number and proportion of fulfilled STROME-ID criteria, which were specified *a priori*. Few studies have specifically examined factors correlated with STROBE reporting quality,[3,4] although this was analyzed using other reporting frameworks (e.g., CONSORT, STARD).[5-7] These include sample size (SS), the journal impact factor (IF), and the geographic region of senior author's primary affiliation. For sample size, the number of patients as well as isolates were extracted from each article. We initially considered sample size both in terms of the number of isolates and number of individual patients. However, Spearman's rho suggested evidence of collinearity between these variables (0·86, P-value <0.01). In light of this, and missing data for the number of patients (n=21 articles, 18·4%), the sample size of isolates was used for further analysis (SS). For IF, this was obtained from *Journal Citation Reports* (https://jcr.clarivate.com) for the year of each article's publication. When IF could not be located in in this database, *SciJournal* (https://scijournal.org) was searched. The continent of the senior author's primary affiliation was determined by examining the geographic region of the last author, which typically represents the senior author in genomic epidemiology as well as other fields.[8,9] When authors had multiple affiliations, the continent from

which the study samples were obtained was assigned as the primary affiliation (Supplemental Table 5). In addition to these, we also included the current h-index (HI) of the senior author. This was obtained using Scopus (https://www.scopus.com).

Statistical Analysis

To assess differences in reporting following STROME-ID's publication, the mean proportions of completed criteria were compared before and after its publication date. A 6-month lag period was included to account for articles that were already in press when STROME-ID was published. Sensitivity analyses were also performed using a 12-month lag period, and excluding articles published 6 and 12 months post-STROME-ID publication. Differences in mean proportions of criteria were compared pre- and post-publication using a two-tailed t-test using R software (version 1.1.456). The least and most reported STROME-ID criteria were also qualitatively assessed to explore differences between periods, excluding criteria that were not eligible for > 20% of articles (Supplemental Figure 1 and 2).

To examine the association between study characteristics and reporting, two main approaches were used. First, we used quasi-Poisson regression (to account for under-dispersion) with the number of criteria completed as the dependent variable. Given not all criteria were applicable across every study, this analysis was restricted to criteria that were applicable across all studies. Second, we used tobit regression (censored between 0 and 1) to assess the association with the *proportion* o criteria that were completed, including all studies in the analysis. The distribution of IFs from all papers is shown in Supplemental Figure 3; IF was used as a categorical variable, with categories chosen based on our experience with the metric, and previous studies that examined correlates with IF.[10,11] For SS, we categorized this into quartiles due to low counts across a wide range of data (Supplemental Figure 4). HI was analyzed as a linear variable.

Variables that had a P-value of $< 0{\cdot}20$ in univariate analyses were included in the final model for each analysis. Pseudo-R^2, the Akaike information criterion, and log-likelihood were calculated to assist with model selection and evaluate fit.

Missing data

The number of patients was missing for 18·4% (n=21) articles. IFs were also not available for articles published during the first year of the journal (2013), which we excluded from further analysis, and from 15 articles published in 2019 (13·16%). To address this, IF was reviewed for all available years to assess the degree of variation. If the variation in IF between years was minor, the most recent value was used (Supplemental Table 2).

Supplemental Results

Themes of the included articles

It is important to note that the themes identified were not mutually-exclusive, i.e., studies could focus on more than one theme. In total, 82 studies used WGS to investigate transmission. When compared to classical genotyping methods, these studies demonstrated WGS' superior ability to identify and confirm epidemiological linkages between different subgroups in an outbreak, and their patterns of transmission.[12-15] This was seen across populations in both high-incidence[15] and low-incidence settings,[16-18] as well as between different groups, such as foreign-born and locally born individuals.[19,20] Authors also found that WGS provided additional resolution to distinguish between recurrent TB cases due to relapse or re-infection.[14,21,22] 36 studies used WGS to examine the evolution of TB and drug resistance. Several studies characterized genomic differences using WGS in order to describe the mechanisms of the microevolution of drug-resistant TB and its transmission.[23-28] Studies also described the evolution of outbreaks in various settings, using WGS to reconstruct the timeline of resistance-conferring mutations.[28-30] Lastly, studies broadly examined the evolution of TB in comparison to human migration patterns.[31-34] Eleven studies used WGS to investigate strains and/or lineages of TB. WGS-based genotyping provided additional resolution to elucidate strain diversity,[35,36] and identify genomic characteristics of different strains.[37,38] WGS was also used to identify strains and the sub-lineages present in a particular region's transmission network.[33,39,40] One of the studies also described a new sub-lineage.[41] Finally, two studies examined associations of TB strains or mutations with clinical outcomes, which included rates of relapse, treatment status, death or loss to follow-up.[22,42]

Association of Study and Journal Characteristics

As a sensitivity analysis, we re-ran univariate and multivariate analyses for quasi-Poisson and tobit regression models separately for manuscripts that were solely classified under the theme of 'transmission' (n=67) and those solely classified under 'evolution' (n=21). Univariate analysis did not identify any associations between reporting and sample size, JIF, HI, or continent of affiliation (data not shown). Multivariate analysis was not conducted due to the reduced number of papers in these strata.

Supplemental Figures

Supplemental Figure 1. Count of "not applicable" papers per STROME-ID criterion, pre-publication.

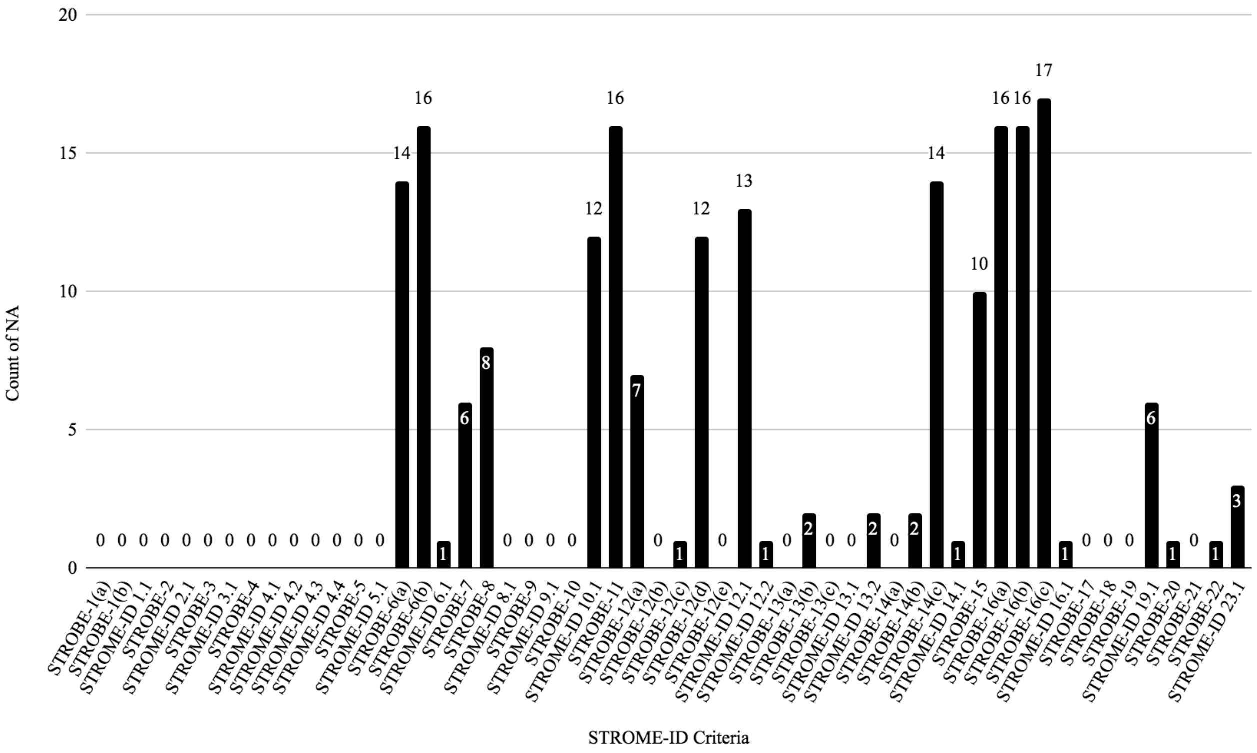

The number of “not applicable” (NA) papers (n= 17) per STROME-ID criterion prior to guideline publication, accounting for a six-month lag. The criterion with the most amount of NA papers required translating estimates of relative risk into absolute risk (STROBE-16c).

Supplemental Figure 2. Count of "not applicable" papers per STROME-ID criterion, post-publication

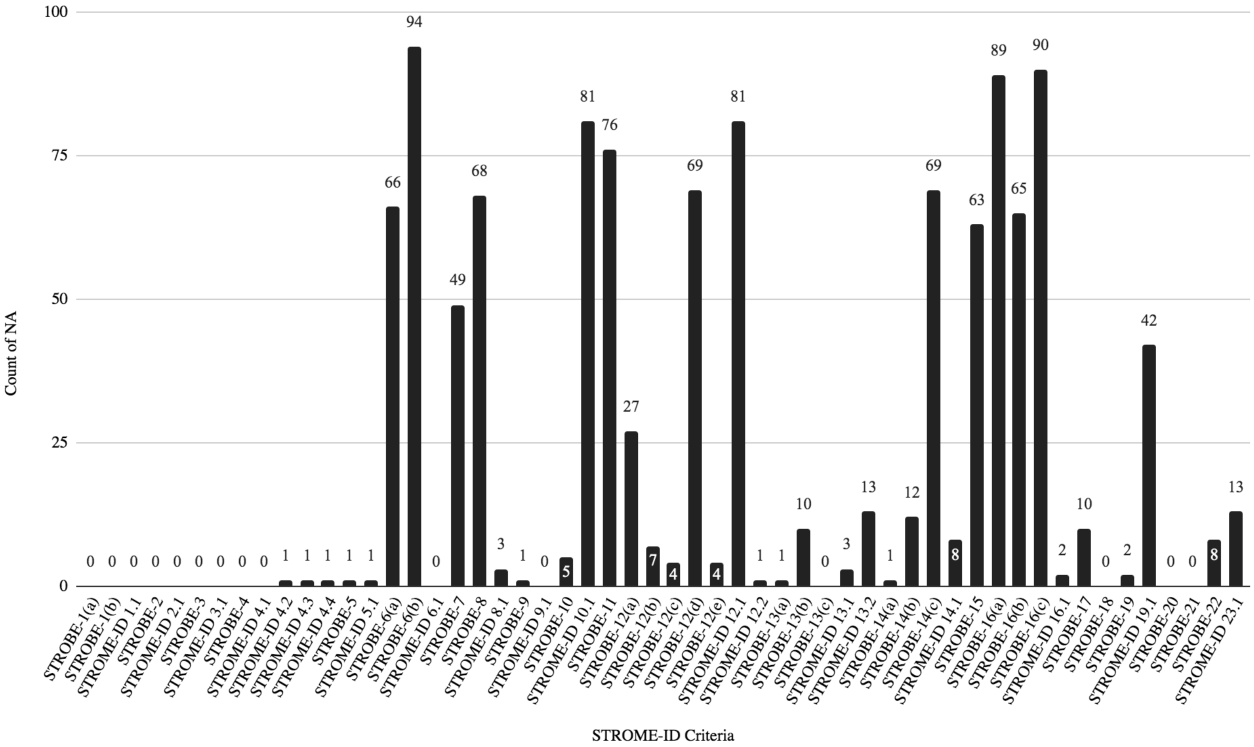

The number of “not applicable” (NA) papers (n= 97) per STROME–ID criterion in the pre-publication reporting period, accounting for a six-month lag. The criterion with the most amount of NA papers required stating the eligibility criteria and methods of participant selection (STROBE-6b).

Supplemental Figure 3. Distribution of impact factors for included papers

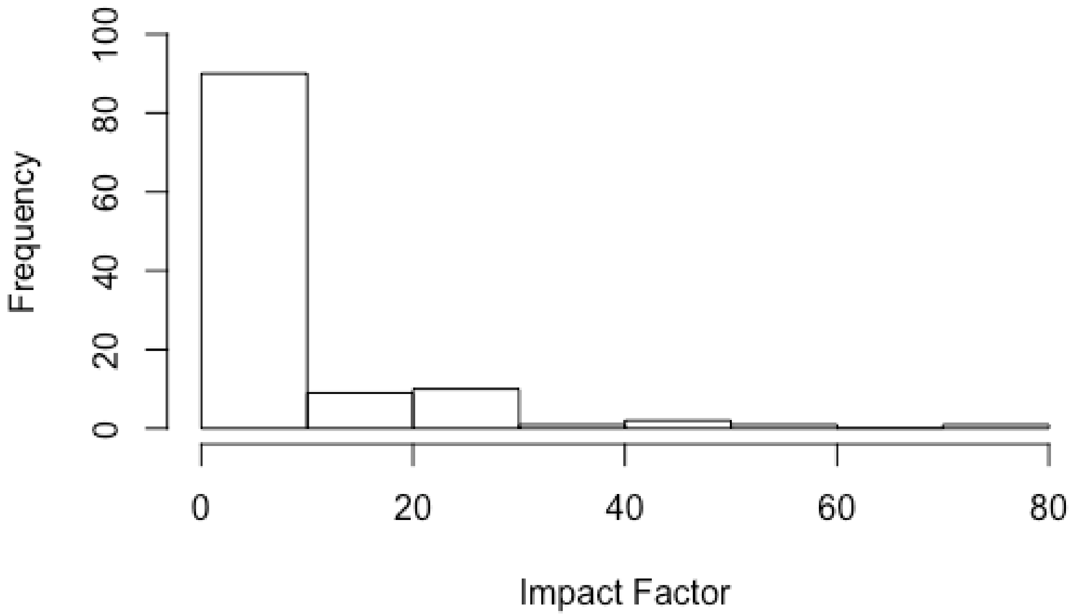

Frequency distribution of journal impact factor (IF). Most IFs in the data set are less than 20, with low counts of IFs greater than 20.

Supplemental Figure 4. Distribution of sample size of isolates in included papers

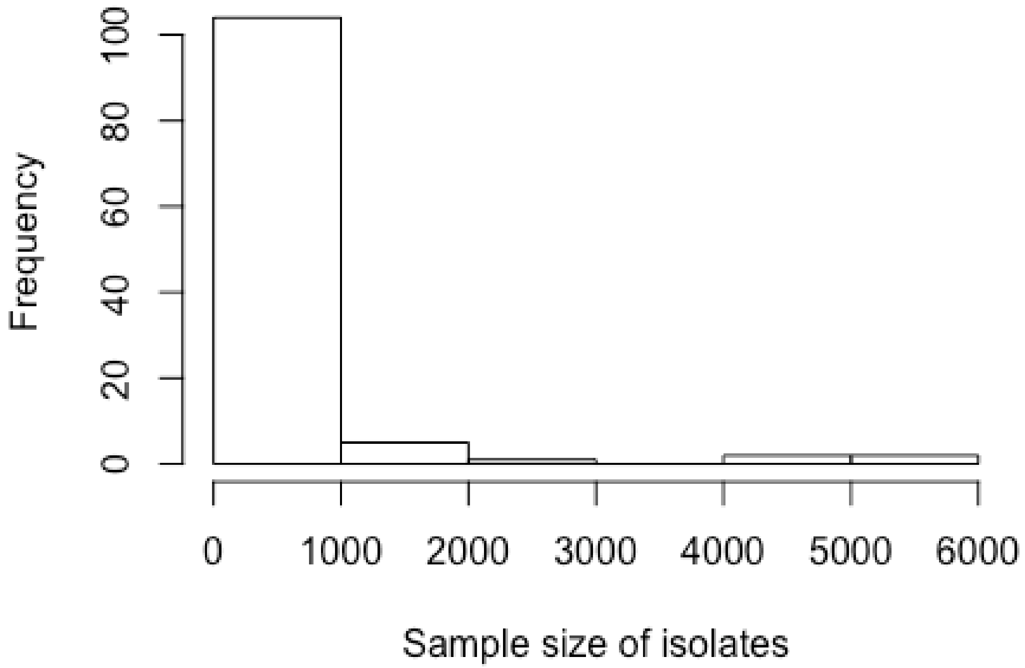

Frequency distribution of sample size of isolates (SS). Most SS are less than 1000, with low counts of SS greater than 1000. There were no counts of SS between 3000 and 4000 isolates.

Supplemental Figure 5. Proportion of STROME-ID criteria met post-publication, excluding articles from the first 6-months following guideline publication.

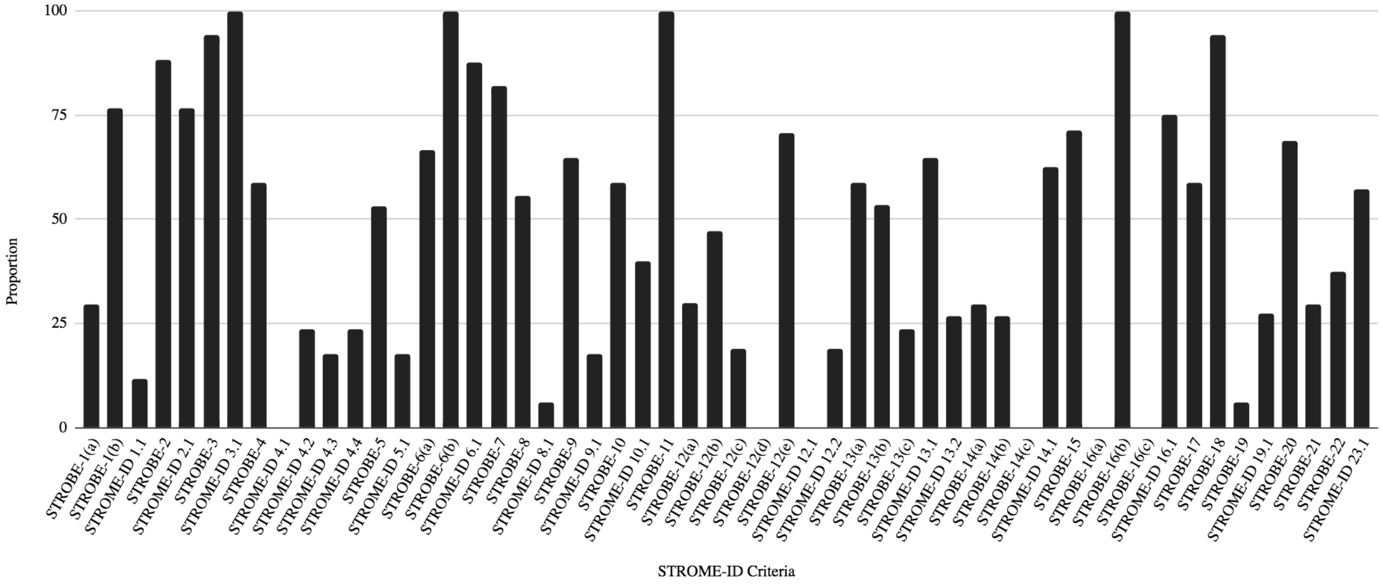

A sensitivity analysis for the time period post-guideline publication was conducted, which excludes articles published in the six months following STROME-ID publication. Six criteria were not completed. This was also observed in the pre-guideline publication period that accounted for the six-month lag. The least completed STROME-ID criterion required definitions for molecular terminology (STROME-ID 4·1). The most completed STROME-ID criterion required stating the epidemiological objectives of using molecular typing (STROME-ID 3·1), the criteria for matched studies (STROBE-6b), the analyses of quantitative variables (STROBE-11), and the category boundaries for categorized variables (STROBE-16b).

Supplemental Figure 6. Proportion of STROME-ID criteria met with 12-month lag pre-publication.

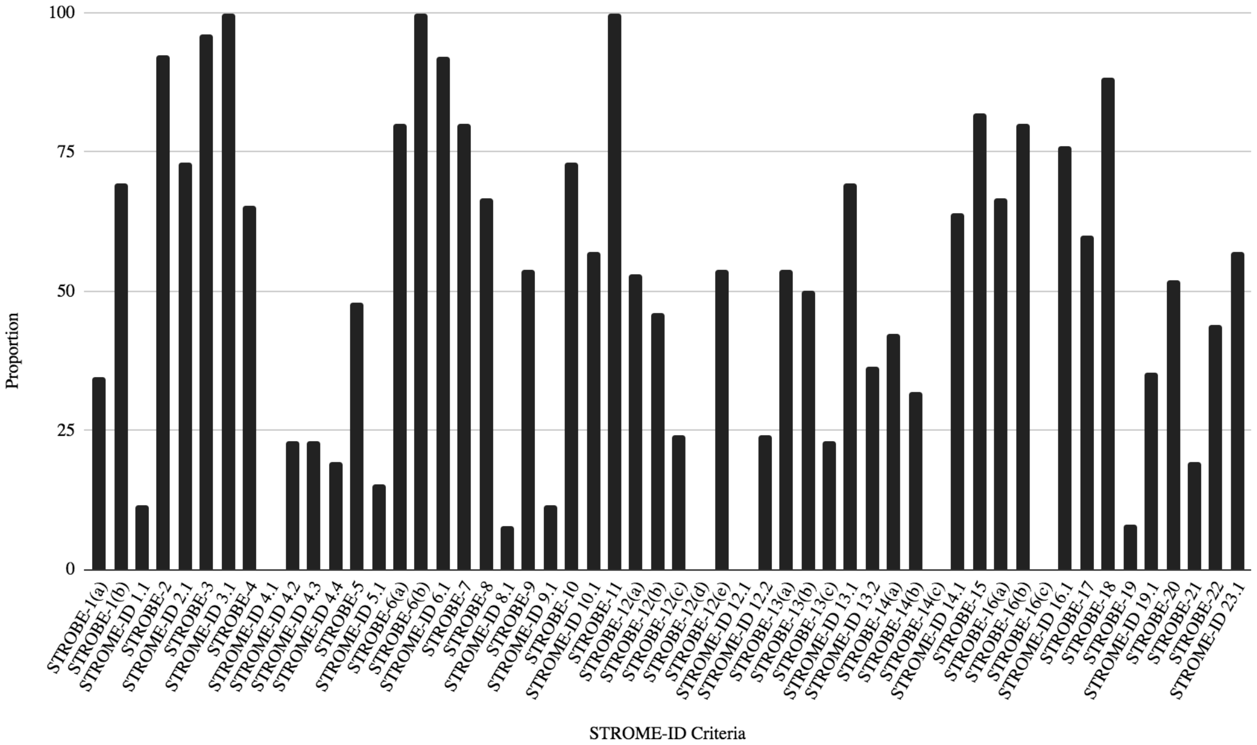

The average proportion of STROME-ID criteria met across articles prior to guideline publication, accounting for a twelve–month lag, was similarly variable to the trend observed in the six-month lag period. The least completed STROME–ID criterion required defining of key molecular terms (STROME-4·1), strategies to address loss to follow-up (STROBE-12d), accounting for the non-independence of data (STROME-ID 12·1), reporting outcome events (STROBE-14c), and translating relative risk into absolute risk (STROBE-16c). The three most frequently reported criteria were: explaining the scientific background and rationale (STROBE–2), stating the epidemiological objectives of using molecular typing (STROME-ID 3·1), and stating study objectives and hypotheses (STROBE-3).

Supplemental Figure 7. Proportion of STROME-ID criteria met with 12-month lag post-publication.

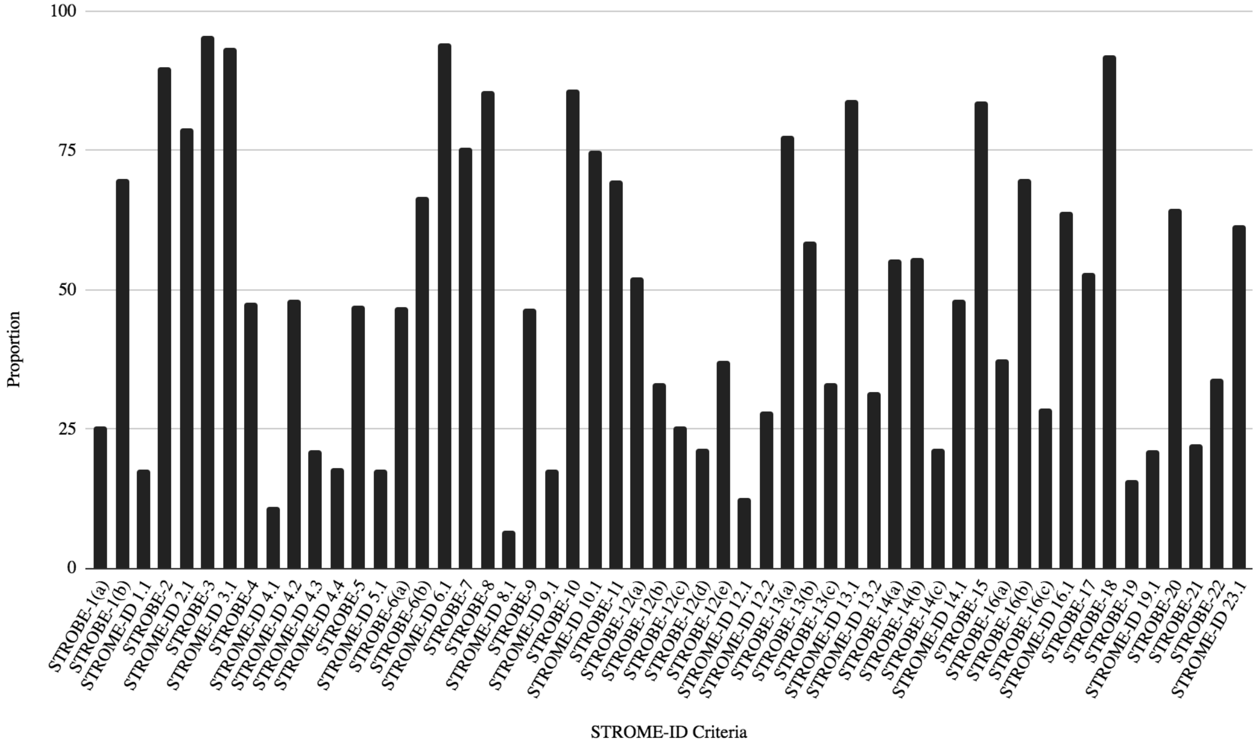

All criteria were completed at least once in this reporting period. The least frequently completed STROME-ID criterion required defining of key molecular terms (STROME-4·1) and describing methods to detect multiple-strain infections (STROME-ID 8·1). The three most frequently completed STROME-ID criteria were: stating the study's overarching objectives and hypotheses (STROBE-3), stating the epidemiological objectives of using molecular typing (STROME-ID 3·1), and stating the source of participants, clinical specimens and the sampling frame (STROME-ID 6·1).

Supplemental Figure 8. Proportion of STROME-ID criteria met post-publication, excluding articles from 12-month lag.

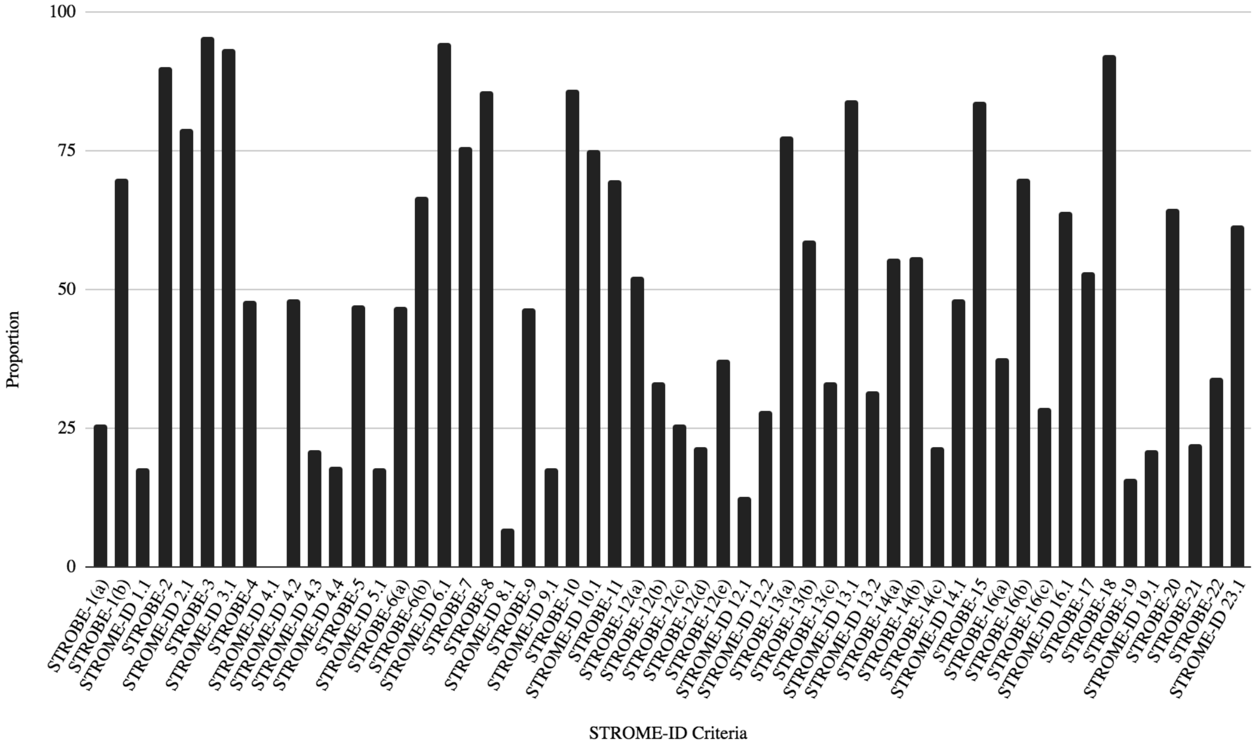

A sensitivity analysis for the time period post-guideline publication was conducted, which excludes articles published in a twelve–month publication lag. Five criteria were not met, which were the same as those criteria not met in the pre-publication period accounting for the 12-month lag.

Supplemental Tables

Supplemental Table 1. STROME-ID criteria, adapted from Field et al.[2]

Criteria	Description
STROBE-1(a)	Denote study's design using a term in the title or the abstract
STROBE-1(b)	Briefly describe methods and results in the abstract
STROME-ID 1.1	The term molecular epidemiology is mentioned in the title or abstract and the keywords
STROBE-2	Describe the scientific context and rationale of the methods used
STROME-ID 2.1	Discuss the pathogen population and the distribution of pathogen strains within the host population
STROBE-3	State study objectives and any prespecified hypotheses
STROME-ID 3.1	State the epidemiological objectives of using molecular typing
STROBE-4	Discuss study design early in the paper
STROME-ID 4.1	Define key molecular terminologies used in the study
STROME-ID 4.2	Define the molecular markers using a standard nomenclature
STROME-ID 4.3	Provide definitions for infectious-disease cases
STROME-ID 4.4	Discuss methods about sample collection, laboratory techniques, and minimizing cross-contamination. Provide criteria for identifying strains
STROBE-5	Provide information about the locations, dates, participant recruitment, exposure, follow-up, and data collection
STROME-ID 5.1	Mention the timeframe of the study. Discuss the molecular clock of markers if known, and its natural history
STROBE-6(a)	*Cohort study*—Provide eligibility criteria, and the sources and methods for including participants. Explain follow-up methods<br>*Case-control study*— Provide eligibility criteria, and the sources and methods of case ascertainment and control selection. Provide explanation for use of cases and controls<br>*Cross-sectional study*— Provide eligibility criteria, and the sources and methods of participant selection
STROBE-6(b)	*Cohort study*—For matched studies, state matching criteria and number of exposed and unexposed participants<br>*Case-control study*—For matched studies, state matching criteria and controls per case
STROME-ID 6.1	Discuss source of participants and clinical specimens. State sampling frame and strategy
STROBE-7	Report all outcomes, exposures, predictors, potential confounders, and effect modifiers. Give diagnostic criteria, if applicable
STROBE-8	For each variable of interest, state data sources and methods of assessment. If more than one group, state comparability of assessment methods.
STROME-ID 8.1	Explain detection of multiple-strain infections
STROBE-9	Explain methods to address potential sources of bias
STROME-ID 9.1	Explain how discovery or ascertainment bias was addressed
STROBE-10	Explain the rationale for study size
STROME-ID 10.1	Report unique restrictions placed on the study sample size
STROBE-11	Describe the analyses of quantitative variables. If relevant, describe rationale for groupings
STROBE-12(a)	Describe all statistical methods, including those used to control for confounding
STROBE-12(b)	Describe any methods used to examine subgroups and interactions
STROBE-12(c)	Discuss methods for addressing missing data
STROBE-12(d)	*Cohort study*—if applicable, explain how loss to follow-up was addressed *case-Control study*—if applicable, explain how matching of cases and controls was addressed<br>*Cross-sectional study*—if applicable, describe analytical methods taking account of sampling strategy
STROBE-12(e)	Describe any sensitivity analyses
STROME-ID 12.1	State how the study took account of the non-independence of sample data, if appropriate
STROME-ID 12.2	Explain methods for addressing missing data

STROBE-13(a)	Discuss count of individuals at each stage of the study
STROBE-13(b)	Provide rationale for non-participation at each stage
STROBE-13(c)	Uses a flow diagram
STROME-ID 13.1	Report numbers of participants and samples at each stage of the study (e.g., number of samples, the number typed, and the number yielding data)
STROME-ID 13.2	If molecular clusters are investigated, report the sampling fraction, cluster sizes, and the study population turnover, if known
STROBE-14(a)	Provide characteristics of study participants, including details about exposures and potential confounders
STROBE-14(b)	Denote the number of individuals with missing data for each variable of interest
STROBE-14(c)	*Cohort study*-summarise follow-up time
STROME-ID 14.1	Give information by strain type if appropriate, with use of standardised nomenclature
STROBE-15	*Cohort study*—state numbers of outcome events or summary measures over time<br>*Case-control study*—state count of each exposure category<br>*Cross-sectional study*—state numbers of outcome events or summary measures
STROBE-16(a)	Provide unadjusted estimates, included confounder-adjusted estimates and their precision if relevant. Explain which confounders were adjusted for and why
STROBE-16(b)	State category boundaries for continuous variables that were categorised
STROBE-16(c)	If relevant, convert relative risk into absolute risk
STROME-ID 16.1	Illustrate molecular similarity among strains with a dendrogram or phylogenetic tree
STROBE-17	Report other analyses done, such as subgroup analyses
STROBE-18	Discuss key results that consider study objectives
STROBE-19	Report limitations, including potential bias or imprecision, and their direction and magnitude.
STROME-ID 19.1	Consider other possible explanations for findings about transmission chains if relevant. State the consistency between molecular and epidemiological evidence
STROBE-20	Discussion of results that consider objectives, limitations, and other studies' results
STROBE-21	Explain the generalizability of study results
STROBE-22	Provide funding sources and their role
STROME-ID 23.1	State ethical considerations and implications for infectious-disease molecular epidemiology

Supplemental Table 2. Standard deviation of journal IF from 2013-2018, shown for the journals corresponding to an article published in 2019.

Journal	SD
BMC Genomics	0·12
BMC Infectious Diseases	0·07
Clinical Infectious Diseases	0·30
Emerging Infectious Diseases	0·46
J Clin Microbiol	0·44
Molecular Ecology	0·20
Nature Scientific Reports	0·58
PLOS One	0·17
Tuberculosis	0·09

IF= impact factor

Supplemental Table 3. Mean proportions of STROME-ID criteria met pre- and post-guideline publication for transmission-only themed papers (n= 67)

Exposure	Pre-STROME-ID (n= 7)	SD	Post-STROME-ID (n= 60)	SD	*P*-value
6 Months	0.54	0.13	0.51	0.12	0.56
12 Months[a]	0.55	0.12	0.51	0.12	0.41
6 Months Exclusion[b]	0.54	0.13	0.51	0.12	0.56
12 Months Exclusion[b]	0.57	0.13	0.51	0.11	0.29

SD= Standard deviation, STROME-ID= Strengthening the Reporting of Molecular Epidemiology for Infectious Diseases
[a]Papers published within 12 months following STROME-ID were classified as 'unexposed', i.e., we considered that authors may not have seen the guidelines or had the opportunity to incorporate them. [b]Papers published in this time period following the STROME-ID publication date were excluded from the analysis altogether.

Supplemental Table 4. Mean proportions of STROME-ID criteria met pre- and post-guideline publication for evolution-only themed papers (n= 21).

Exposure	Pre-STROME-ID (n= 7)	SD	Post-STROME-ID (n= 14)	SD	*P*-value
6 Months	0.38	0.14	0.48	0.11	0.10
12 Months[a]	0.38	0.14	0.48	0.11	0.10
6 Months Exclusion[b]	0.36	0.14	0.48	0.11	0.10
12 Months Exclusion[b]	0.39	0.13	0.49	0.11	0.07

SD= Standard deviation, STROME-ID= Strengthening the Reporting of Molecular Epidemiology for Infectious Diseases
[a]Papers published within 12 months following STROME-ID were classified as 'unexposed', i.e., we considered that authors may not have seen the guidelines or had the opportunity to incorporate them. [b]Papers published in this time period following the STROME-ID publication date were excluded from the analysis altogether

Supplemental Table 5. Count of papers per continent of senior author's primary affiliation.

Continent of senior author's primary affiliation	Count of papers
North America	32
South America	1
Africa	6
Asia	13
Europe	54
Oceania	8

Note: Due to low individual country counts, countries were grouped by continent, where South America was included with North America for the category "Americas" because it had only one count.

Supplemental Table 6. Univariate and multivariate tobit analysis of IF, HI, Continent, and SS.

	Univariate			Multivariate		
Variables	Coefficients	95% CI	*P*-value	Coefficients	95% CI	*P*-value
IF						
0-4.9999*						
5-9.9999	0·05	-0·0003, 0·10	0·05	0·04	-0·02, 0·09	0·16
10-19.9999	0·08	0·006, 0·16	0·04	0·06	-0·02, 0·14	0·14
≥20	0·10	0·03, 0·16	0·003	0·06	-0·01, 0·12	0·09
HI	0·0002	-0·001, 0·001	0·80			
Continent						
Americas *†						
Africa	0·04	-0·06, 0·14	0·40	0·03	-0·06, 0·12	0·54
Asia	-0·04	-0·11, 0·04	0·31	-0·03	-0·10, 0·04	0·34
Europe	-0·04	-0·09, 0·01	0·15	-0·04	-0·09, 0·01	0·08
Oceania	-0·04	-0·13, 0·05	0·41	-0·005	-0·01, 0·08	0·91
SS						
<30*						
30-152	0·07	0·01, 0·12	0·02	0·05	0·0004, 0·11	0·05
153-276	0·09	0·02, 0·16	0·02	0·07	-0·01, 0·14	0·07
≥277	0·11	0·05, 0·17	< 0·0001	0·09	0·02, 0·15	0·01

*Reference level
†Combined North America and South America; only 1 country from South America
CI= confidence interval; HI= h-index ; IF= impact factor; IRR= incidence rate ratio; SS= sample size of isolates

Supplemental Table 7. Sensitivity univariate and multivariate analysis for quasi-Poisson, excluding twelve papers with >1 senior author.

	Univariate			Multivariate		
**Variables**	**IRR**	**95% CI**	***P*-value**	**IRR**	**95% CI**	***P*-value**
IF						
0-4·9999*						
5-9·9999	1·11	1·00, 1·23	0·03	1·10	0·99, 1·23	0·07
10-19·9999	1.19	1·02, 1·38	0·03	1·15	0·97, 1·36	0·11
≥20	1·18	1·00, 1·37	0·04	1·12	0·94, 1·32	0·21
HI	1·00	1·00, 1·00	0·33			
Continent						
Americas *†						
Africa	0·99	0·80, 1·21	0·93	1·01	0·82, 1·24	0·92
Asia	0·92	0·79, 1·08	0·31	0·95	0·81, 1·11	0·54
Europe	0·91	0·82, 1·01	0·07	0·91	0·82, 1·00	0·05
Oceania	0·93	0·77, 1·11	0·42	1·00	0·83, 1·21	0·97
SS						
<30*						
30-152	1·02	0·91, 1·14	0·75	1·00	0·89, 1·11	0·92
153-276	1·04	0·88, 1·21	0·65	1·01	0·86, 1·19	0·90
≥277	1·16	1·03, 1·31	0·02	1·10	0·96, 1·26	0·16

*Reference level
†Combined North America and South America; only 1 country from South America
CI= confidence interval; HI= h-index; IF= impact factor; IRR= incidence rate ratio; SS= sample size of isolates

Supplemental Table 8. Sensitivity univariate and multivariate analysis for tobit regression, excluding twelve papers with >1 senior author.

	Univariate			Multivariate		
**Variables**	**Coefficient**	**95% CI**	***P*-value**	**Coefficient**	**95% CI**	***P*-value**
IF						
0-4.9999*						
5-9.9999	0·04	0.00, 0·10	0·09	0·03	-0·02, 0·08	0·24
10-19.9999	0·07	-0·02, 0·15	0·12	0·03	-0·05, 0·12	0·42
≥20	0·08	-0·01, 0·16	0·07	0·03	-0·05, 0·14	0·49
HI	0·0002	-0·001, 0·001	0·69			
Continent						
Americas *†						
Africa	0·04	-0·06, 0·15	0·41	0·03	-0·07, 0·14	0·50
Asia	-0·04	-0·12, 0·03	0·27	-0·04	-0·11, 0·04	0·32
Europe	-0·05	-0·10, 0·00	0·07	-0·05	-0·10, 0·00	0·05
Oceania	-0·05	-0·14, 0·04	0·31	-0·004	-0·09, 0·09	0·93
SS						
<30*						
30-152	0·07	0·01, 0·12	0·01	0·06	0·00, 0·11	0·04
153-276	0·06	-0·02, 0·13	0·15	0·05	-0·03, 0·13	0·20
≥277	0·11	0·05, 0·17	< 0·0001	0·10	0·04, 0·17	0·002

*Reference level
†Combined North America and South America; only 1 country from South America
CI= confidence interval; HI= h-index; IF= impact factor; IRR= incidence rate ratio; SS= sample size of isolates

Supplemental Table 9. Number of papers with unavailable raw genomic data.

Publication year	Papers with unavailable raw genomic data	Total papers
2009	0	1
2010	1	2
2011	0	1
2012	0	1
2013	0	9
2014	0	6
2015	3	18
2016	2	12
2017	4	17
2018	14	34
2019	4	13

References

1. Liberati A, Altman DG, Tetzlaff J, et al. The PRISMA statement for reporting systematic reviews and meta-analyses of studies that evaluate health care interventions: explanation and elaboration. *Ann Intern Med* 2009; **151**(4): W65-W94.
2. Field N, Cohen T, Struelens MJ, et al. Strengthening the reporting of molecular epidemiology for infectious diseases (STROME-ID): an extension of the STROBE statement. *Lancet Infect Dis* 2014; **14**(4): 341-52.
3. Rao A, Brück K, Methven S, et al. Quality of reporting and study design of CKD cohort studies assessing mortality in the elderly before and after STROBE: a systematic review. *PLoS ONE* 2016; **11**(5): e0155078.
4. Adams AD, Benner RS, Riggs TW, Chescheir NC. Use of the STROBE checklist to evaluate the reporting quality of observational research in obstetrics. *Obstet Gynecol* 2018; **132**(2): 507-12.
5. Ghimire S, Kyung E, Lee H, Kim E. Oncology trial abstracts showed suboptimal improvement in reporting: a comparative before-and-after evaluation using CONSORT for abstract guidelines. *J Clin Epidemiol* 2014; **67**(6): 658-66.
6. Selman TJ, Morris RK, Zamora J, Khan KS. The quality of reporting of primary test accuracy studies in obstetrics and gynaecology: application of the STARD criteria. *BMC Women's Health* 2011; **11**(1): 8.
7. Mackinnon S, Drozdowska BA, Hamilton M, Noel-Storr AH, McShane R, Quinn T. Are methodological quality and completeness of reporting associated with citation-based measures of publication impact? A secondary analysis of a systematic review of dementia biomarker studies. *BMJ Open* 2018; **8**(3): e020331.
8. Bhopal R, Rankin J, McColl E, et al. The vexed question of authorship: views of researchers in a British medical faculty. *BMJ* 1997; **314**(7086): 1009-12.
9. D Reisenberg GL. The order of authorship: who's on first? *JAMA* 1990; (264): 1857.
10. Kuroki LM, Allsworth JE, Peipert JF. Methodology and analytic techniques used in clinical research: associations with journal impact factor. *Obstet Gynecol* 2009; **114**(4): 877-84.
11. Falagas ME, Kouranos VD, Michalopoulos A, Rodopoulou SP, Batsiou MA, Karageorgopoulos DE. Original artile: comparison of the distribution of citations received by articles published in high, moderate, and low impact factor journals in clinical medicine. *Intern Med J* 2010; **40**(8): 587-91.
12. Jajou R, de Neeling A, van Hunen R, et al. Epidemiological links between tuberculosis cases identified twice as efficiently by whole genome sequencing than conventional molecular typing: A population-based study. *PLoS One* 2018; **13**(5).
13. Ocheretina O, Shen L, Escuyer VE, et al. Whole genome sequencing investigation of a tuberculosis outbreak in Port-au-Prince, Haiti caused by a strain with a "low-level" rpoB mutation L511P - insights into a mechanism of resistance escalation. *PLoS one* 2015; **10**(6): e0129207.
14. Witney AA, Bateson AL, Jindani A, et al. Use of whole-genome sequencing to distinguish relapse from reinfection in a completed tuberculosis clinical trial. *BMC Med* 2017; **15**(1): 71.
15. Wyllie D, Davidson J, Walker T, et al. A quantitative evaluation of MIRU-VNTR typing against whole-genome sequencing for identifying Mycobacterium tuberculosis transmission: a prospective observational cohort study. *EBioMedicine* 2018; **34**: 122-30.
16. Cabibbe AM, Trovato A, De Filippo MR, et al. Countrywide implementation of whole genome sequencing: an opportunity to improve tuberculosis management, surveillance and contact tracing in low incidence countries. *The Eur Respir J* 2018.
17. Gurjav U, Outhred AC, Jelfs P, et al. Whole genome sequencing demonstrates limited transmission within identified Mycobacterium tuberculosis clusters in New South Wales, Australia. *PLoS ONE* 2016; **11**(10): e0163612.
18. Genestet C, Tatai C, Berland JL, et al. Prospective whole-genome sequencing in tuberculosis outbreak investigation, France, 2017-2018. *Emerg Infect Dis* 2019; **25**(3): 589-92.
19. Auld SC, Shah NS, Mathema B, et al. Extensively drug-resistant tuberculosis in South Africa: genomic evidence supporting transmission in communities. *Eur Respir J* 2018; **52**(4).
20. Mizukoshi F, Miyoshi-Akiyama T, Iwai H, et al. Genetic diversity of Mycobacterium tuberculosis isolates from Tochigi prefecture, a local region of Japan. *BMC Infect Dis* 2017; **17**(1): 365.
21. Bryant JM, Harris SR, Parkhill J, et al. Whole-genome sequencing to establish relapse or re-infection with Mycobacterium tuberculosis: a retrospective observational study. *Lancet Respir Med* 2013; **1**(10): 786-92.
22. Guerra-Assunçao JA, Houben RMGJ, Crampin AC, et al. Recurrence due to relapse or reinfection with Mycobacterium tuberculosis: a whole-genome sequencing approach in a large, population-based cohort with a high HIV infection prevalence and active follow-up. *J Infect Dis* 2015; **211**(7): 1154-63.
23. Perez-Lago L, Comas I, Navarro Y, et al. Whole genome sequencing analysis of intrapatient microevolution in Mycobacterium tuberculosis: potential impact on the inference of tuberculosis transmission. *J Infect Dis* 2014; **209**(1): 98-108.
24. Casali N, Nikolayevskyy V, Balabanova Y, et al. Microevolution of extensively drug-resistant tuberculosis in Russia. *Genome Res* 2012; **22**(4): 735-45.
25. Kato-Maeda M, Ho C, Passarelli B, et al. Use of whole genome sequencing to determine the microevolution of Mycobacterium tuberculosis during an outbreak. *PLoS ONE* 2013; **8**(3): e58235.

26. Mehaffy C, Guthrie JL, Alexander DC, Stuart R, Rea E, Jamieson FB. Marked microevolution of a unique Mycobacterium tuberculosis strain in 17 years of ongoing transmission in a high risk population. *PLoS ONE* 2014; **9**(11): 0112928.
27. Ioerger TR, Koo S, No E-G, et al. Genome analysis of multi- and extensively-drug-resistant tuberculosis from KwaZulu-Natal, South Africa. *PLoS ONE* 2009; **4**(11): e7778.
28. Cohen KA, Abeel T, Manson McGuire A, et al. Evolution of Extensively Drug-Resistant Tuberculosis over Four Decades: Whole Genome Sequencing and Dating Analysis of Mycobacterium tuberculosis Isolates from KwaZulu-Natal. *PLoS Medicine* 2015; **12**(9): e1001880.
29. Ioerger TR, Feng Y, Chen X, et al. The non-clonality of drug resistance in Beijing-genotype isolates of Mycobacterium tuberculosis from the Western Cape of South Africa. *BMC Genomics* 2010; **11**: 670.
30. Casali N, Broda A, Harris SR, Parkhill J, Brown T, Drobniewski F. Whole genome sequence analysis of a large isoniazid-resistant tuberculosis outbreak in london: a retrospective observational study. *PLoS Medicine* 2016; **13**(10): e1002137.
31. Eldholm V, Monteserin J, Rieux A, et al. Four decades of transmission of a multidrug-resistant Mycobacterium tuberculosis outbreak strain. *Nat Commun* 2015; **6**.
32. Comas I, Coscolla M, Luo T, et al. Out-of-Africa migration and Neolithic coexpansion of Mycobacterium tuberculosis with modern humans. *Nat Genet* 2013; **45**(10): 1176-82.
33. Luo T, Comas I, Luo D, et al. Southern East Asian origin and coexpansion of Mycobacterium tuberculosis Beijing family with Han Chinese. *Proc Natl Acad Sci U S A* 2015; **112**(26): 8136-41.
34. O'Neill MB, Shockey A, Zarley A, et al. Lineage specific histories of Mycobacterium tuberculosis dispersal in Africa and Eurasia. *Mol Ecol* 2019; **28**(13): 3241-56.
35. Stucki D, Brites D, Jeljeli L, et al. Mycobacterium tuberculosis lineage 4 comprises globally distributed and geographically restricted sublineages. *Nat Genet* 2016; **48**(12): 1535-43.
36. Chatterjee A, Nilgiriwala K, Saranath D, Rodrigues C, Mistry N. Whole genome sequencing of clinical strains of Mycobacterium tuberculosis from Mumbai, India: A potential tool for determining drug-resistance and strain lineage. *Tuberculosis* 2017; **107**: 63-72.
37. Gautam SS, Mac Aogain M, Bower JE, Basu I, O'Toole RF. Differential carriage of virulence-associated loci in the New Zealand Rangipo outbreak strain of Mycobacterium tuberculosis. *Infect Dis* 2017; **49**(9): 680-8.
38. Koster K, Largen A, Foster JT, et al. Whole genome SNP analysis suggests unique virulence factor differences of the Beijing and Manila families of Mycobacterium tuberculosis found in Hawaii. *PLoS ONE* 2018; **13**(7).
39. Casali N, Nikolayevskyy V, Balabanova Y, et al. Evolution and transmission of drug-resistant tuberculosis in a Russian population. *Nat Genet* 2014; **46**(3): 279-86.
40. Winglee K, Manson McGuire A, Maiga M, et al. Whole genome sequencing of Mycobacterium africanum strains from Mali provides insights into the mechanisms of geographic restriction. *PLoS Negl Trop Dis* 2016; **10**(1): e0004332.
41. Malm S, Linguissi LSG, Tekwu EM, et al. New Mycobacterium tuberculosis complex sublineage, Brazzaville, Congo. *Emerg Infect Dis* 2017; **23**(3): 423-9.
42. Sobkowiak B, Glynn JR, Houben R, et al. Identifying mixed Mycobacterium tuberculosis infections from whole genome sequence data. *BMC Genomics* 2018; **19**(1): 613.
